# Ionic Plasticity: Common Mechanistic Underpinnings of Pathology in Spinal Cord Injury and the Brain

**DOI:** 10.3390/cells11182910

**Published:** 2022-09-17

**Authors:** Kelsey E. Hudson, James W. Grau

**Affiliations:** 1 Neuroscience, Texas A&M University, College Station, TX 77843, USA; 2 Psychological & Brain Sciences, Texas A&M University, College Station, TX 77843, USA

**Keywords:** spinal cord injury, GABA, ionic plasticity, pain, spasticity, epilepsy, addiction, KCC2

## Abstract

The neurotransmitter GABA is normally characterized as having an inhibitory effect on neural activity in the adult central nervous system (CNS), which quells over-excitation and limits neural plasticity. Spinal cord injury (SCI) can bring about a modification that weakens the inhibitory effect of GABA in the central gray caudal to injury. This change is linked to the downregulation of the potassium/chloride cotransporter (KCC2) and the consequent rise in intracellular Cl^−^ in the postsynaptic neuron. As the intracellular concentration increases, the inward flow of Cl^−^ through an ionotropic GABA-A receptor is reduced, which decreases its hyperpolarizing (inhibitory) effect, a modulatory effect known as ionic plasticity. The loss of GABA-dependent inhibition enables a state of over-excitation within the spinal cord that fosters aberrant motor activity (spasticity) and chronic pain. A downregulation of KCC2 also contributes to the development of a number of brain-dependent pathologies linked to states of neural over-excitation, including epilepsy, addiction, and developmental disorders, along with other diseases such as hypertension, asthma, and irritable bowel syndrome. Pharmacological treatments that target ionic plasticity have been shown to bring therapeutic benefits.

## 1. Introduction

The neurotransmitter gamma-aminobutyric acid (GABA) is traditionally characterized as having an inhibitory effect on neural activity within the adult central nervous system (CNS) [1]. This inhibition places a brake on neural excitability and plasticity. At a functional level, this helps to preserve neural circuits over time and prevent seizure activity. This classic view informs medical pharmacology and motivates the development of therapeutics designed to augment GABAergic inhibition to quell neural over-excitation.

Here, we review the mechanisms that mediate GABA’s effect on neural activity, with a focus on the ionotropic GABA-A receptor. Engaging this receptor allows the anion chloride (Cl^−^) to flow across the neural membrane. Under normal conditions, the intracellular concentration of Cl^−^ in the adult central nervous system (CNS) is low and, for this reason, engaging the GABA-A receptor allows the anion to flow into the cell which has a hyperpolarizing (inhibitory) effect [2]. Research has shown that spinal cord injury (SCI) can induce a modification in the central gray caudal to injury that causes a rise in the intracellular concentration of Cl^−^. This reduces the inward flow of Cl^−^, which lessens the inhibitory effect of engaging the GABA-A receptor and removes a brake on neural activity, allowing a state of over-excitation that can drive aberrant motor activity (spasticity) and foster the sensitization of pain (nociceptive) circuits in the spinal cord dorsal horn [3,4].

**Figure 1:**
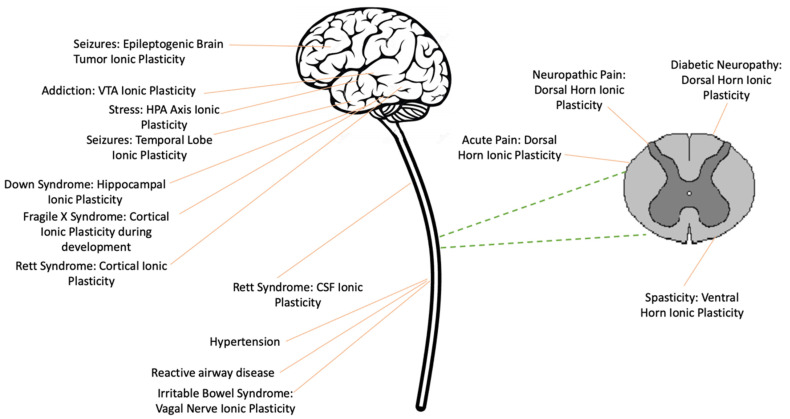
Ionic plasticity along the neural axis. A pictorial representation of the neural axis indicating the locations of identified ionic plasticity covered in this review and the associated neural disease states.

In the following sections, we introduce key concepts and describe how an alteration in intracellular Cl^−^ can reduce GABA-dependent inhibition, a phenomenon known as *ionic plasticity* [5]. We then describe how this process enables a state of over-excitation within the spinal cord and how ionic plasticity contributes to long-term pathology (Figure 1).

Finally, we discuss evidence that these same processes contribute to a number of brain-dependent pathologies (Figure 1). The work suggests new treatments that aim to re-establish GABAergic inhibition by targeting the processes that underlie ionic plasticity.

## 2. Foundations: GABA, Ionic Plasticity, and KCC2

The current review will focus on the phenomenon of ionic plasticity, which impacts neural excitability by altering the flow of the anion Cl^−^ through the GABA-A receptor. To understand how this concept has transformed our views of neural processing, and how the inhibitory effect of GABA is regulated, we begin with an overview of the GABA receptors, describing their alternative forms and pharmacological agents that impact GABAergic function. We then describe how the effect of engaging the GABA-A receptor depends upon the intracellular concentration of Cl^−^, how this is regulated by membrane-bound co-transporters, and how this process can be studied using drugs that target these co-transporters. While subsequent sections will touch on studies that have used genetic techniques to explore these processes, our focus will be on research employing pharmacological tools that can be readily translated to the clinic.

### 2.1. Regulation of Neural Excitability

The central nervous system continuously integrates excitatory and inhibitory signals. This balance allows important neural signals to be processed without being lost in excessive static neuronal activity. The major inhibitory neurotransmitter in the mature CNS is GABA. Its functions span the CNS, including the cerebral cortex, amygdala, hippocampus, and spinal cord [6]. GABA can also be found in peripheral tissues, though its role in these regions is less well understood. GABA-specific neurons can be either long-range signaling neurons or shorter-acting interneurons. GABA is also found in neurons that primarily signal using other neurotransmitters. GABAergic activity varies based on numerous factors: the extent of synaptic connectivity, the volume and activity of enzymes that produce GABA, the degree of excitatory drive to the GABAergic neurons themselves, the density of GABA receptors present, the presence of GABA scavengers, and other neurological modulators [7].

GABA functions via three receptor systems; GABA-A, GABA-B, and GABA-C. GABA-B and GABA-C, though important, are less prevalent than GABA-A. GABA-B receptors are metabotropic GABA receptors that mediate the long-term, slow inhibitory actions of GABA. GABA-B receptors are members of the class-C family of G-protein coupled receptors [8]. Operating as heterodimers, the two subunits come together to form a functioning receptor that activates various G-proteins. GABA-B receptors are linked to potassium channels, adenylate cyclase, and calcium channels through their G-proteins. This class of receptors is always inhibitory [9]. Their metabotropic signaling mechanisms can drive potassium efflux, decrease calcium conductance, and inhibit cAMP production, all of which inhibit neural excitation. These receptors mediate both presynaptic and postsynaptic inhibition and are largely limited to the spinal cord [1].

GABA-C receptors are ionotropic. They are highly restricted, found primarily in the retina, and only account for a minute fraction of the total GABA receptors [8]. GABA-C receptors are very similar to GABA-A receptors but have some unique pharmacological properties [9].

The predominant receptors, GABA-A, are ionotropic and allow for the flow of anions, primarily Cl^−^, down their concentration gradients [8]. This receptor subtype is responsible for the fast responses to GABA, reacting on the millisecond time scale. GABA-A receptors are found in 20–50% of synapses in the brain [6]. They are a member of the pentameric ligand-gated ion channel superfamily. Functioning GABA-A receptors are formed by five subunits coming together to form a central anion-permeable core [6]. A total of 19 different subunits have been identified [6]. These assemble in limited combinations of five, yielding distinct forms of the receptor. The predominant isoform of the GABA-A receptor in the human adult brain is α_1_β_2_γ_2_ [6]. The various combinations of subunits have different global and temporal expression patterns, subcellular targeting, kinetics, and pharmacological properties. The binding of two GABA molecules is required to induce the conformational change that opens the channel and allows anions to flow through the pore.

Several cellular processes can influence the degree to which engaging a GABAergic neuron affects post-synaptic neural activity, including the subunit structure of the GABA-A receptor and the trafficking of GABARs [9]. In addition, the availability of GABA for release and its duration of action are impacted by its synthesis by glutamic acid decarboxylase (GAD), packaging by vesicular transporter (VGAT), and reuptake by GABA transporters (GATs) [7]. For further discussion of how these processes regulate GABA function and may contribute to pathology, see [7,10,11,12,13]. Here, we focus on a downstream process, the regulation of ion flow through the GABA-receptor. In the following section, we discuss how this process can be targeted using pharmacological agents. We then describe cellular processes that can alter the flow of Cl^−^ by modulating its intracellular concentration and how these mechanisms can be pharmacologically targeted.

### 2.2. GABA Receptor: Pharmacology

**Figure 2:**
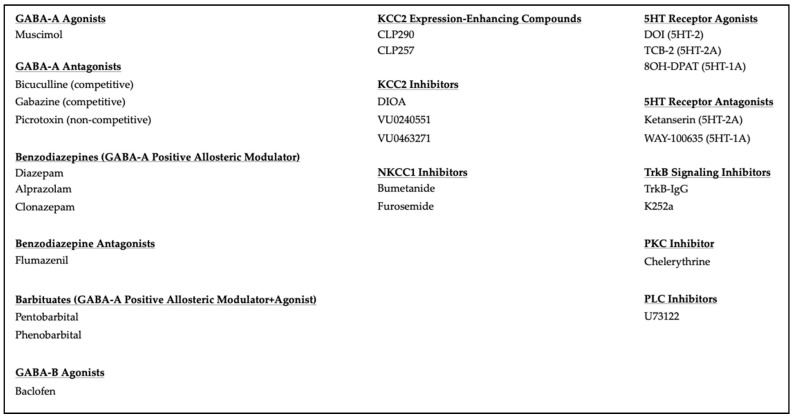
Drugs used to target GABA functionality and ionic plasticity.

When addressing the clinical potential of a neurological phenomenon, it is necessary to examine the tools available to modulate it. Fortunately, there is a long history of research on GABA function, supported by a host of pharmacological tools (Figure 2). Many are GABA-A selective and although they are among the most successful clinical drugs they are also common substances of abuse [14].

In general, treatments that engage GABA receptors produce “downer” effects. They have anticonvulsant, anti-anxiety, sedative, analgesic, and anesthetic properties, all of which stem from the increased inhibitory tone that they induce [15]. These can act in one of two ways. Drug agonists emulate key structural features of GABA and bind to the same receptor site with varying affinity. Muscimol is a naturally occurring GABA analog that engages the GABA-A receptor [9]. The GABA agonist baclofen has a high affinity for GABA-B receptors and is used primarily as a muscle relaxer [16].

The second manner by which GABAergic drugs can act is through allosteric modulation that amplifies Cl^−^ conductance when the GABA-A receptor is engaged by GABA [15]. Benzodiazepines bind to sites on the alpha subunits of the GABA-A receptor and act as positive allosteric modulators potentiating the action of endogenous GABA release. Drugs in this class have a sedative effect and include diazepam, alprazolam, and clonazepam. Benzodiazepine use is extremely common. In fact, over the course of a single year, nearly 13% of adults in the United States were prescribed benzodiazepines [14].

Barbituates, such as phenobarbital and pentobarbital, also function as positive allosteric modulators but bind to sites on the beta subunits of GABA-A receptors. At high concentrations, these drugs can also act as GABA agonists [1]. Barbituates increase the proportion of GABA channels in the longest open state and reduce the proportion of channels in the shortest open state, thus prolonging the mean channel open time and increasing Cl^−^ flux [9]. Barbituates can also impact glutamatergic AMPA receptors, reducing membrane depolarization and neuronal excitability. While a benzodiazepine (e.g., midazolam) may enhance the potency of GABA two-fold, pentobarbital has an effect that augments potency 20-fold. Drugs in this class are frequently used to induce an anesthetic state for surgery and at high doses can produce electroencephalographic silence (barbiturate coma) and fatal respiratory depression [1].

GABA-A receptor activity can be blocked with bicuculline, which is a competitive antagonist at the GABA binding site. It is widely used in research for its convulsant properties and acts by decreasing the opening frequency and mean open time of the GABA-A channel, thus decreasing Cl^−^ flux [9]. Picrotoxin is a plant-derived alkaloid that disrupts GABA-A function in a non-competitive manner (e.g., by blocking the anion channel). It has a diverse history as a central nervous stimulant and performance-enhancing drug [15]. However, its toxicity limits its use [9]. Flumazenil is a GABA-A receptor antagonist that acts specifically on the benzodiazepine active site of the receptor. As such, it is used clinically to treat benzodiazepine overdose [17]. As with GABA agonists, there are many other lesser-used GABA antagonists.

### 2.3. Regulation of Intracellular Cl^−^ Concentrations

Recent research has revealed that engaging the GABA-A receptor can have varied effects on the adult CNS [18]. It is now understood that, unlike GABA-B receptors which are always inhibitory, the effects of GABA-A receptor activation can be inhibitory or excitatory depending on the state of ionic balance, particularly the Cl^−^ concentration gradient. Changes in the relative concentration gradient of Cl^−^ ions regulate the inhibitory/excitatory gradient of GABA-A activity, drastically altering the effects of GABA release, a phenomenon termed ionic plasticity [19].

#### 2.3.1. Co-Transporters KCC2 and NKCC1

Ion transporters and pumps maintain the concentration gradients between intracellular and extracellular compartments throughout the body. In the CNS, the sodium-potassium-chloride cotransporter (NKCC1) and the potassium-chloride cotransporter (KCC2) are the major players responsible for Cl^−^ homeostasis [20]. NKCC1, an ion transporter from the NKCC family, is one of the main Cl^−^ intruders. During transport, it transfers one sodium ion, one potassium ion, and two Cl^−^ ions into the cell. Given that water follows the flow of ions, NKCC1 is a key regulator of cell volume. In early development, NKCC1 expression is high, controlling the Cl^−^ balance with its dominant activity [21]. The expression of NKCC1 decreases in postnatal development but is maintained at moderate levels in mature CNS neurons [20].

KCC2 is one of four isoforms of KCC ion transporters. All KCCs extrude a single Cl^−^ ion in exchange for one potassium ion. While KCC1, KCC3, and KCC4 have been found throughout the nervous system, their expression levels are negligible in neurons, and their roles are not clearly elucidated. In numerous other tissues, their functions are better understood. KCC2 is the only Cl^−^ extruder in most CNS neurons [20]. KCC2 is expressed abundantly in the mature nervous system, with little-to-no expression in the peripheral nervous system or non-neuronal cell types. In fact, KCC2 is the only KCC isoform not expressed in glia [22]. The temporal expression profile of KCC2 is very different from that of NKCC1. KCC2 expression is low in early development and increases later, eventually reaching the point where KCC2 activity becomes predominant in maintaining Cl^−^ homeostasis [21].

While GABA is the major inhibitory neurotransmitter in the mature CNS, it functions as an excitatory neurotransmitter in the early phases of development. Research demonstrates that both states of GABA are important for nervous system development and normal function later. The specific timing of this switch varies from species to species and across CNS regions, generally paralleling the maturation of the nervous system [21].

Ultimately, the shift in GABA functionality (ionic plasticity) is the consequence of the changing expression profiles of NKCC1 and KCC2 throughout development. In early development, NKCC1 expression is high while KCC2 expression is relatively low. In the human cortex, NKCC1 peaks from post-conceptional week 31 to 41. At the same time, KCC2 expression is only 2–25% of adult levels [23]. As such, Cl^−^ concentrations are high intracellularly. Thus, when GABA activates GABA-A receptors, Cl^−^ flows out of the cell, driving depolarizing currents and leading to excitation of the neurons in question. This process is highly non-linear; even small changes in intracellular Cl- concentration can change the direction of ion flow [24].

Enabling anion flow and engaging the GABA-A receptor can also have a current shunting effect that undermines the capacity for an excitatory input to reach threshold and evoke an action potential, which reduces neuronal firing. This shunting effect may dominate GABA-A receptors positioned on the soma [2]. In contrast, engaging dendritic GABA-A receptors when intracellular concentrations of Cl- are increased will have a depolarizing effect that propagates to the soma. Finally, there is variation in the propensity for shifts in KCC2 across areas of the nervous system. For example, noxious stimulation more readily induces a depolarizing shift in lamina I of the superficial dorsal horn, relative to lamina II [25,26].

As the CNS matures, the expression of NKCC1 decreases while KCC2 expression increases [22]. This drives the intracellular concentration of Cl^−^ down, to one-tenth of the extracellular concentration in the mature nervous system. Maintaining such low intracellular Cl^−^ is essential for GABA’s inhibitory functionality. In this state, when GABA activates GABA-A receptors, there is a rapid influx of Cl^−^ ions that leads to hyperpolarization, inhibiting the affected neurons.

**Figure 3:**
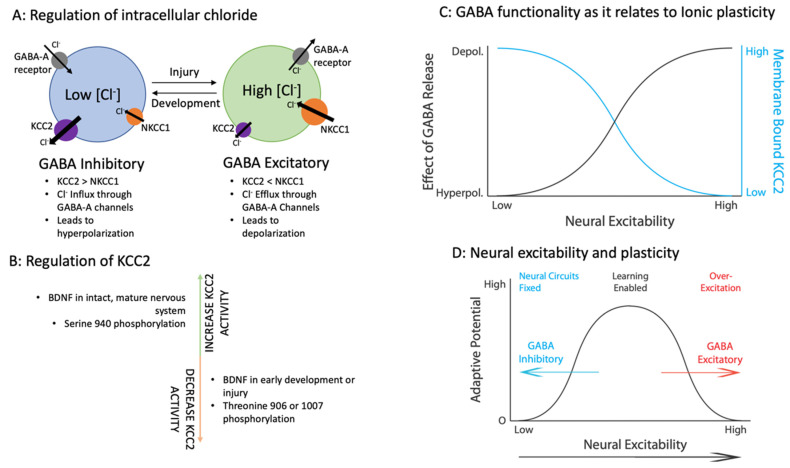
Overview of ionic plasticity. (**A**) Regulation of intracellular Cl^−^. Figure depicting the relative functional levels of KCC2: NKCC1, the direction of Cl^−^ flux through GABA-A channels, and resultant effects of GABA activity on the cell. (**B**) Regulation of KCC2. Demonstrating the major effectors that increase and decrease KCC2 functionality. (**C**) GABA functionality as it relates to ionic plasticity. Demonstrating the inverse relation between KCC2 activity and GABA functionality. Adapted from: [19]. (**D**) Neural excitability and plasticity. Theorized relationship between the state of ionic plasticity and the state of neural plasticity. Adapted from: [19].

The drastic differences in ionic homeostasis and the flip of these concentration gradients have been understood in neurodevelopment for some time. While these shifts are key to healthy neural development, ionic plasticity is now recognized in the mature nervous system as well (Figure 3) [19]. The traditional view of a largely rigid mature nervous system has changed as the potential scope of neuroplasticity has grown. In particular, the mature nervous system demonstrates significant plasticity after insult or injury. Because ionic plasticity impacts plastic potential in excitatory circuits, we [19] and others [26] have suggested that this plasticity of plasticity represents a form of metaplasticity [27].

It must be acknowledged that some controversy has emerged regarding the hypothesized developmental shift in GABA function. At issue is whether this represents a general effect, with in vivo evidence that GABA may retain some inhibitory action early in development (see Valeeva et al. 2016 and Kirmse et al. 2015) [28,29]. Yet even those who doubt aspects of the developmental hypothesis acknowledge that injury and disease can bring a shift in intracellular Cl^−^ concentration that impacts GABA function, which is the focus of the present review. Furthermore, many of the pathological effects do not require a flip in GABA function to excitation. Instead, simply lessening a GABA-dependent brake on neural activity may be sufficient to enable glutamatergic over-excitation and pathology.

Ionic plasticity has now been identified in many pathologies, allowing the nervous system to revert to a state with greater plasticity. The results of this shift may yield adaptive plasticity allowing for improved recovery, learning, or repair. Or it may cause maladaptive plasticity yielding pain, malfunction, over-excitation, and further damage. The continuum of ionic plasticity (Figure 3D) suggests that there might be an ideal “happy medium” where adaptive plasticity can be fostered, and maladaptive plasticity avoided. While the potential benefits of this transformation will be noted, our focus will be on how this modification can lead to pathology and the implications for treatment.

#### 2.3.2. Cellular Processes That Regulate Ionic Plasticity

As described above, the Cl^−^ balance in the CNS is maintained by KCC2 and NKCC1. Their expression levels are controlled by various signals including brain-derived neurotrophic factor (BDNF), insulin-like growth factor (IGF), and cystic fibrosis transmembrane conductance regulator (CFTR) [21]. The specific subunit composition, membrane stability, and the oligomeric or monomeric state of the cation-chloride transporter also play roles in NKCC1 and KCC2 functionality [30]. NKCC1 and KCC2 activity is further regulated by phosphorylation through numerous enzymes whose activity can be modulated by top-down signaling; hormones, such as estrogen, oxytocin, and taurine; and other signals [20,21]. While the regulation of cation-chloride cotransporters is vast, several regulatory factors are integral to the state of ionic plasticity in the mature nervous system. These include BDNF signaling, the phosphorylation of KCC2, and neuronal stress.

BDNF is a neurotrophin with many important functions. Beyond its role in neuronal survival and differentiation, BDNF directly controls neural activity and synaptic plasticity [31]. The regulation of BDNF signaling cascades is complicated by numerous receptor isoforms that drive a multitude of responses varying in time and location. BDNF impacts glutamatergic signaling and its role in GABAergic signaling has recently become a focus of research. It has been suggested that the activity-dependent regulation of BDNF expression may account for variation in ionic plasticity across neural regions [26].

**Figure 4:**
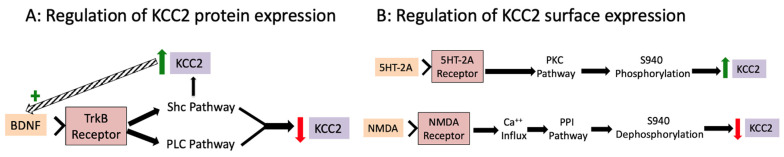
This figure highlights several key cellular mechanisms that regulate KCC2 function. While the complexity of regulating the key players of ionic plasticity (KCC2 and NKCC1) is yet to be fully elucidated, we highlight here key concepts relevant to the current review. (**A**) Regulation of KCC2 protein expression. Figure depicting the pathways by which BDNF regulates KCC2 protein expression. (**B**) Regulation of KCC2 surface expression. Figure depicting two known signaling pathways that regulate KCC2 surface expression and stability by means of altering the phosphorylation state of KCC2. Adapted, in part, from: [30].

The activity of BDNF in GABA signaling and ionic plasticity is context dependent. Regulation of KCC2 by BDNF differs depending on the developmental stage as well as the integrity of the nervous system. In an intact mature nervous system, BDNF downregulates KCC2 expression [31]. In an immature nervous system, or after injury, BDNF can have the opposite effect, upregulating KCC2 expression [32]. Interestingly, both occur by activating the tropomyosin-receptor-kinase-B (TrkB) signaling cascade (Figure 4A). This cascade has multiple paths with different effectors. It is now understood that these are active at different times and likely drive the varying functions of BDNF-TrkB signaling [33]. Two important effectors for the regulation of KCC2 by BDNF are phospholipase C-γ (PLCγ) and Src homologous and collagen-like protein (Shc). In the uninjured adult CNS both PLCγ and Shc are activated when BDNF binds to TrkB. The co-activation of PLCγ and Shc drives the downregulation of KCC2. PLCγ is downregulated in early development, as well as after SCI and other neurological damage. In the absence of PLCγ, only the Shc portion of the BDNF-TrkB cascade is activated [33]. Alone, Shc-mediated activation drives BDNF to upregulate KCC2. This dichotomous activity explains the divergent effects of BDNF administration on neurological pathologies such as nociceptive sensitization and spasticity (reviewed below).

BDNF is expressed in various neuronal cell types, astrocytes, and microglia. Microglia are a predominant source of BDNF in multiple neurological disorders [31]. Various extracellular signals from inflammation, injury, or other insults increase the expression of P2X4R, a purinergic receptor found on microglia. When tissue is damaged, stored adenosine-5-triphosphate (ATP) is released into the extracellular space and acts on the P2X4R receptor triggering microglia to release BDNF [31]. This BDNF then acts on the BDNF-TrkB cascade and alters KCC2, shifting the ionic balance.

Another key regulator of KCC2 activity is its phosphorylation state, which can bring about a change in membrane-bound KCC2 activity within minutes [34]. Phosphorylation at critical residues affects the cell surface stability, intrinsic ion transport rate, and plasmalemmal trafficking of KCC2, all of which alter its Cl^−^ extrusion capacity. KCC2 activity is bi-directionally controlled by its phosphorylation state [35]. If phosphorylated on serine 940 (S940), or key tyrosine residues, KCC2 surface expression and function are enhanced [36,37]. This phosphorylation is mediated by protein kinase C (PKC). While several signaling cascades drive PKC activity, its activation through descending serotonergic (5HT) pathways and serotonin-2A (5HT-2A) receptors in the spinal cord is of particular interest regarding ionic plasticity (Figure 4B). Dephosphorylation of S940 has the opposite effect, decreasing KCC2 surface expression. Interestingly, high levels of NMDA activation drive calcium influx which activates protein phosphatase 1 (PP1) resulting in dephosphorylation of S940, decreasing KCC2 activity (Figure 4B) [30]. Functionally, this would remove a brake on neural activity during periods of strong neuronal drive, reducing the metabolic load. In contrast to serine 940 phosphorylation, if KCC2 is phosphorylated on threonines 906 (T906) and/or 1007 (T1007), its activity is inhibited [36]. Phosphorylation of KCC2 acts rapidly and explains the fast time scale (minutes to hours) of activity-dependent functional changes in KCC2 and ionic plasticity [35].

Given the numerous regulators of KCC2, and thus ionic plasticity, Wake and colleagues examined the effects of neurological stress on KCC2. Across three methods of experimentally induced neuronal stress (oxidative stress, induced seizure activity, and placing neurons in a hyperexcitable state), all produced rapid dephosphorylation of tyrosine residues on KCC2 [37], which reduced functional KCC2 activity. The shift in the phosphorylation state occurred rapidly followed by decreases in protein and mRNA expression. These results demonstrate that neurological stressors induce a biphasic reduction of KCC2 functionality: (1) dephosphorylation leads to a functional downregulation and (2) a downregulation of KCC2 protein levels [37]. Because the maintenance of a low intracellular Cl- concentration requires metabolic energy, allowing the collapse of this gradient during periods of neuronal stress could help to preserve energy stores and neuronal survival [5,20].

Psychological stress too can drive ionic plasticity in various brain regions and in the hypothalamic-pituitary-adrenal axis, the body’s stress system itself [38,39]. Furthermore, maternal stress can decrease KCC2 expression in offspring [40]. Conversely, environmental enrichment and exercise can promote KCC2 expression [40,41,42].

#### 2.3.3. Pharmacology That Regulates Ionic Plasticity

A number of pharmacological agents have been shown to affect the cation-chloride cotransporters responsible for maintaining GABA-A inhibition (Figure 2). These provide therapeutic potential without inducing some of the pitfalls associated with drugs that act at GABA receptors, such as sedation, decreased respiratory drive, and addictive potential [14]. The most well-studied drug is bumetanide, a loop diuretic that has been used to treat edema and hypertension. Its activity as an NKCC1 antagonist has provided a tool to study ionic plasticity, acting to reduce the inward flow of Cl^−^, thereby reducing its intracellular concentration [43]. Furosemide is a diuretic commonly used to treat hypertension and heart failure. Like bumetanide, it impacts sodium-potassium-chloride transport. It has an inhibitory effect on NKCC1 at low concentrations and KCC2 at high concentrations [44]. Recently, CLP257 and CLP290 (the carbamate prodrug of CLP257) were identified. These are KCC2 selective agonists with significant therapeutic potential [45]. In addition, there are numerous studies evaluating pre-existing FDA-approved drugs for efficacy on the cation-chloride cotransporters as well as large-scale screenings of new compounds [46].

### 2.4. Summary

Recent work has led to a new view of GABA that recognizes its effect on neural activity is dynamically regulated. What has transformed our views of GABA function is the recognition that cellular systems can alter the hyperpolarizing effect of engaging the GABA-A receptor, removing a brake, and enabling neural excitability/plasticity. In the sections that follow, we describe how this modification fuels the development of spasticity and pain after SCI. We then discuss how ionic plasticity and the consequent over-excitation contribute to several brain-dependent pathologies. In all cases, the link to ionic plasticity suggests new therapeutic options.

## 3. Spasticity

The emergence of GABA-dependent inhibition within the spinal cord parallels the development of descending fibers from the brain. Indeed, cutting the spinal cord at birth has been reported to block the hyperpolarizing shift in GABA function in the caudal central gray (Jean-Xavier et al., 2006). Likewise, SCI in adult animals causes a downregulation in membrane-bound KCC2 that attenuates the inhibitory effect of GABA. As described below, this loss of inhibition enables aberrant motor activity (spasticity) and impairs the recovery of motor function after SCI.

### 3.1. Clinical Symptoms and Indices

When considering the effects of disrupting the balance of excitatory and inhibitory signaling in the nervous system, spasticity is the foremost disease state that comes to mind. Spasticity is defined by the irregular stiffening or tightening of muscles that prevents normal, fluid movement. It can affect any type of movement in any muscle. As a result, spasticity can limit or even prevent everyday activities such as gait, feeding oneself, or speech [47]. Spasticity is typically caused by damage to the spinal cord or brain, which drives an imbalance of the excitatory and inhibitory signals that the muscles receive. The nervous system damage that drives spasticity can result from a traumatic brain injury, an SCI, or other injuries. After SCI, more than 80% of patients experience spasticity, and approximately half of those patients require medication for its treatment [48]. Spasticity may also be caused by an ischemic insult such as a stroke. While stroke symptoms are dependent upon the brain region affected, around 30% of patients experience spasticity in some part of their body [49]. Other neurological diseases such as cerebral palsy, amyotrophic lateral sclerosis, and multiple sclerosis can also cause spasticity.

Spasticity can be detected and quantified using electrophysiological methods that examine the vigor of the Hoffmann reflex (H-reflex). The H-reflex is an electrical correlate of the stretch reflex and indicates the level of excitability of alpha motor neurons [50]. There are several measures related to the H-reflex that are indicative of spasticity. First and foremost, rate-dependent depression [RDD; also known as frequency-dependent depression (FDD)] is an electrophysiological measure well correlated with the degree of spasticity in patients. RDD refers to the decline in H-reflex amplitude with repeated stimulation, a habituation-like effect that has been linked to GABAergic inhibition. The development of RDD is slowed with spasticity, and for this reason, H-reflex amplitude remains strong [50]. Another measure of spasticity is the H-reflex threshold or the lowest stimulation level that can elicit an H-reflex response. The threshold of the H-reflex decreases with increased excitability, and lower H-reflex thresholds are indicative of spasticity [50]. Clinically, these electrophysiological measures are often superseded by behavioral indicators of spasticity. Patients present with contractures, lack of fluidity, muscle stiffness, involuntary movements, and pain or discomfort [47].

Despite the numerous patient populations affected by spasticity, treatment options are still limited, and those that do exist are often ineffective. Fortunately, physical therapy has consistently shown beneficial results. Despite the successes, treatment with physical therapy is limited due to time, resources, financial limitations, and the fact that the benefits often depend upon continuous treatment that is difficult to sustain [51]. Pharmacological treatments, on the other hand, are marred by significant and common side effects. Available drugs that target GABA often lead to sedation, long-lasting depression of spinal neuronal activity, and dizziness. Furthermore, the agents have an addictive quality. These secondary effects can interfere with everyday life and aspects of the recovery process [52].

Evidence suggests that spasticity arises from increased motor neuron excitability and increased synaptic inputs in response to muscle stretch [47]. This is, at least in part, due to a loss of GABA-dependent inhibition. As we will see below, the process that mediates this transformation is ionic plasticity.

### 3.2. Reduced GABAergic Inhibition Enables Spasticity

Evidence that ionic plasticity plays a pivotal role in the development and maintenance of spasticity was provided by Boulenguez and colleagues [3]. They first examined the expression of the major Cl^−^ transporters, NKCC1 and KCC2. They found that NKCC1 expression was not altered after SCI, but KCC2 was significantly reduced [3]. These results were consistent across injury models (transection or contusion) and age (adult or early postnatal rats) [3]. They also showed that KCC2 immunostaining was reduced in the ventral horn after SCI [3]. Electrophysiology demonstrated that synaptic inhibition was decreased significantly as a result of the altered state of Cl^−^ [3]. These results provide evidence that SCI alters Cl^−^ homeostasis and that this impacts neural excitability.

Additionally, Boulenguez and colleagues assessed H-reflex parameters. They verified that motor neuron activity was increased in KCC2-deficient mice and by pharmacological inhibition of KCC2 using ((dihydronindenyl)oxy) alkanoic acid (DIOA) [3]. The H-reflex threshold and H-reflex RDD were both significantly decreased. These results were replicated in SCI mice [3]. The downregulation of KCC2 drives both a decrease in inhibition and RDD, both of which are considered essential for the development of spasticity. These results provide evidence that ionic plasticity is, indeed, a key component of spasticity after SCI.

Application of BDNF had a restorative effect that up-regulated plasmalemmal KCC2 and RDD of the H-reflex [3]. This is consistent with other work showing that axotomy or damage to corticospinal neurons can shift how BDNF affects KCC2, causing it to increase expression [53]. In the case of spasticity, this alteration has been linked to a loss of serotonergic fibers that engage the 5HT-2A receptor [54].

Toda et al., 2014 showed that spinally-mediated ionic plasticity also plays a role in post-stroke spasticity. They first confirmed that their model of stroke produces spasticity and that KCC2 is decreased in the spinal cord of stroke subjects [55]. This KCC2 downregulation was sided [55]. That is, KCC2 was downregulated on the ipsilateral side of the stroke in experimental animals when compared to the contralateral side or either side of sham-operated animals. While KCC2 downregulation was confirmed at three- and seven-days post-injury, KCC2 levels normalized by day forty-two post-injury [55]. This suggests that ionic plasticity is necessary for the induction of spasticity, but once established, the maintenance of spasticity no longer depends upon downregulated KCC2. Furthermore, the phosphorylation of KCC2 at serine 940 was decreased on the stroke-affected side three days post-injury [55]. This phosphorylation change would yield early downregulation of KCC2 activity. Toda et al., 2014 also assessed NKCC1 and found that its levels in the spinal cord were unchanged after stroke [55]. These results suggest that ionic plasticity occurs in the spinal cord after stroke and that these changes are important for the induction of post-stroke spasticity, but not its maintenance.

It is important to recognize that not all instances of spasticity are coupled to a downregulation of KCC2 and ionic plasticity. An example is provided by the neurodegenerative disease amyotrophic lateral sclerosis (ALS), which leads to muscle cramps, twitching, and stiffness. Mòdol et al., 2014 used a genetic model of ALS to examine whether these effects are related to a downregulation of KCC2. For a positive control, they also evaluated the effect of SCI and motor axotomy, both of which reduced KCC2. [56]. However, KCC2 levels were not altered in the lumbar spinal motor neurons of ALS model animals. This is particularly remarkable because ALS induces neuronal damage and cell death in a way that mirrors numerous other nerve injury models yet does not drive KCC2 downregulation. Spasticity in ALS, therefore, is driven by a separate mechanism and does not appear to be dependent on ionic plasticity.

### 3.3. Restoring GABAergic Inhibition Attenuates Spasticity

#### 3.3.1. Pharmacotherapeutics for Spasticity

The mounting evidence that spasticity secondary to neurological insults is tied to downregulation of KCC2 has motivated the evaluation of pharmacotherapies that target ionic plasticity. Given that the downregulation of KCC2 after SCI is related to a loss of descending 5HT fibers [3,57], Bos et al., 2013 assessed the therapeutic potential of 5HT agonists [54]. They found that 2,5-dimethoxy-4-iodoamphetamine hydrochloride (DOI), a non-selective serotonin-2 receptor agonist, caused hyperpolarization of the inhibitory postsynaptic potential following SCI, restoring values to levels similar to those in uninjured controls. They also found that KCC2 was significantly increased in the membrane-bound fraction. Furthermore, KCC2 immunostaining was returned to levels comparable to uninjured controls following chronic treatment with DOI [54]. To further clarify the 5HT pathway involved, Bos and colleagues used (4-bromo-3.6-dimethoxybenzovyclobuten-1-yl)methylamine hydrobromide (TCB-2), a high-affinity 5HT-2A receptor agonist. They found that TCB-2 induced hyperpolarization of the inhibitory post-synaptic potential similar to that of DOI, in both injured and uninjured animals [54]. KCC2 expression was also increased following treatments with TCB-2 [54]. To confirm that the TCB-2-induced restoration of inhibitory postsynaptic potentials was dependent upon KCC2 changes, they co-administered VU0240551, a potent KCC2 blocker. As expected, this prevented the TCB-2-induced restoration of hyperpolarization of inhibitory postsynaptic potentials [54].

Bos and colleagues went on to examine the second messenger pathway involved in the restoration of KCC2 and inhibitory post-synaptic potential hyperpolarization through 5HT-2A receptor activation. Knowing that 5HT-2A activation stimulates PLC, which activates protein kinase C (PKC), they assessed the effects of the PKC inhibitor chelerythrine [54]. Chelerythrine prevented both DOI and TCB-2-induced hyperpolarization [54]. Conversely, activation of PKC with phorbol 12,13-dibutyrate (PDBu) induced hyperpolarization of inhibitory postsynaptic potentials similar to those seen with TCB-2 and DOI [54]. Therefore, activation of the 5HT-2A receptor appears to restore KCC2 function, at least in part, by PKC-dependent phosphorylation of KCC2.

Finally, to assess the efficacy of 5HT activation on spasticity symptomology, Bos and colleagues evaluated H-reflex parameters in animals with SCI. As expected, TCB-2 significantly increased RDD of the H-reflex and this effect was blocked by co-administration of the KCC2 antagonist VU0240551 [54]. TCB-2 also decreased the amplitude of the H-reflex in animals with established spasticity [54]. Both of these results demonstrate the efficacy of 5HT-2A agonists in the treatment of spasticity following SCI.

More recently, Bilchak and colleagues tested the therapeutic efficacy of restoring KCC2 function with CLP257 [41]. They found that administration of CLP257 to the lumbar enlargement of the spinal cord restored H-reflex modulation [41]. Bilchak et al. went on to demonstrate effects with several other measures of hyperreflexia and spasticity. They showed that CLP257 also reduces phasic and tonic muscle responses as well as reflex-mediated force [41]. To assess the mechanism behind the behavioral effects of CLP257 administration, Bilchak and colleagues examined KCC2 expression. They found that CLP257 administration restored membrane expression of KCC2 in the lumbar motoneurons [41]. Together, these results demonstrate that CLP257 does decrease spasticity after chronic SCI and that these effects are likely the result of increased KCC2 expression and the resultant restoration of GABAergic inhibition.

#### 3.3.2. Exercise a Spasticity Therapeutic

The ability of rehabilitative exercise to alleviate spasticity is accepted based on clinical success [58]. However, the mechanisms that underlie these improvements were unknown. Using an animal model, Tashiro et al., 2015 confirmed that treadmill training attenuated spasticity, limiting it to almost baseline levels [42]. They also demonstrated that BDNF expression was increased with treadmill training along with phosphorylation of TrkB, both of which would increase signaling in that molecular pathway [42]. To assess the direct effects of treadmill training on ionic plasticity, Tashiro and colleagues then assessed KCC2 expression. They found that the ratio of phosphorylated (active) KCC2 to total KCC2 was increased by treadmill training [42]. These results correlated with their measures of spasticity. Overall, KCC2 immunoreactivity in lamina I/II, as well as immunostaining of the plasma membranes of motor neurons for KCC2, was increased by treadmill training [42]. To discern whether the effect of treadmill training on KCC2 was mediated by BDNF signaling, they administered TrkB-immunoglobulin (TrkB-IgG) to inhibit the signaling cascade. Animals that received TrkB-IgG did not exhibit the behavioral benefits of treadmill training, nor did they have increased KCC2 levels [42].

Given the evidence that exercise increases both BDNF and KCC2 levels in the spinal cord after SCI, Beverungen and colleagues used electrophysiological measures of spasticity to evaluate the therapeutic potential of treadmill training and the role of ionic plasticity [59]. They first verified that rehabilitation prevents the decrease in H-reflex threshold after SCI. Moreover, blocking KCC2 activity with VU0240551 blocked the effect of exercise on H-reflex threshold [59]. Thus, the exercise-induced rescue of H-reflex threshold required KCC2. FDD, another electrophysiological correlate of spasticity, was improved in animals that were exercised, and again, VU0240551 administration prevented activity-dependent recovery of FDD [59]. What is more, blocking KCC2 activity during therapeutic sessions also prevented the activity-dependent decrease in the Hmax/Mmax ratio, yet another indication that KCC2 activity is essential for exercise to decrease spinal hyperexcitability [59].

Beverungen and colleagues next assessed the necessity of BDNF signaling in the therapeutic benefits of exercise on spasticity. They found that administration of TrkB-IgG yielded a pattern of results that mirrored those found with VU0240551 [59]. Blocking BDNF activity prevented rehabilitation from reducing spinal hyperexcitability. Furthermore, blocking KCC2 or BDNF did not affect hyperreflexia in unexercised animals [59]. Therefore, therapy is inducing an increase in KCC2 and BDNF that is prevented by blocking BDNF activity.

At a cellular level, Bevernugen et al. confirmed that KCC2 expression is decreased after SCI [59]. Importantly, this downregulation was prevented by their exercise program. The restoration of KCC2 expression was maintained whether or not KCC2 activity was inhibited during rehabilitation [59]. Yet, if BDNF activity was inhibited, the exercise-induced elevation of KCC2 expression was prevented. In addition, the exercise program prevented a BDNF downregulation [59]. In addition, blocking KCC2 activity reduced BDNF levels, preventing the exercise-induced increase [59]. This suggests a circular modulatory effect wherein BDNF regulates KCC2 and KCC2, in turn, regulates BDNF.

Beverungen et al., 2020 also assessed NKCC1. Given its opposing role to KCC2, they examined whether rehabilitation affected its activity and KCC2 in opposite ways. They found that SCI increased NKCC1 expression and demonstrated that exercise also prevented this effect [59]. Blocking KCC2 activity with VU0240551 prevented the exercise-dependent decrease of NKCC1. Inhibiting BDNF, on the other hand, did not affect NKCC1 expression levels [59]. This suggests that NKCC1 and KCC2 are reciprocally regulated and both likely contribute to exercise-induced changes in spinal excitability.

Collectively, the results of Beverungen and colleagues confirm that ionic plasticity alters spinal excitability following SCI. The results also demonstrate that rehabilitation exercise programs reverse ionic plasticity and restore KCC2, NKCC1, and BDNF levels. These molecular changes, in turn, reduce motor neuron excitability and alleviate the symptoms of spasticity.

Li and colleagues examined the mechanistic underpinnings of successful treatment through treadmill training [60]. Like Beverungen et al., Li et al. demonstrated that exercise improves the RDD of the H-reflex and the Hmax/Mmax ratio, both indicators of spasticity. Li et al. found that their exercise paradigm, body weight-supported treadmill training, increased both BDNF and TrkB expression [60]. To assess the necessity of these changes in the therapeutic efficacy of their exercise program, Li and colleagues blocked BDNF signaling with TrkB-IgG. TrkB-IgG administration prevented the exercise-induced reduction in spasticity and increase in KCC2 expression [60]. Together, these results suggest ionic plasticity drives the development of spasticity after SCI and demonstrate therapeutic mechanisms to target for treatment.

### 3.4. Ionic Plasticity Disrupts the Recovery of Motor Function

Other recent findings suggest that alterations in GABA function can affect the recovery of motor function after SCI independent of spasticity. This was discovered by Chen and colleagues in a screening of drugs that impact recovery in animals that have undergone a dual-hemisection [61]. In this model, animals receive two lateral hemisections that end on the midline on opposite sides at different segments (T7 and T10). Together, the two hemisections cut all ascending and descending fibers between the brain and lumbosacral spinal cord. Over time, animals recover some capacity for hindlimb locomotion, which has been attributed to adaptive rewiring in the intervening tissue, providing a functional bridge. A small molecule screen of drugs known to affect neuronal excitability revealed that the KCC2 activator CLP290 promoted the recovery of locomotor function. Bumetanide too had a positive effect, but this did not reach statistical significance [61].

Given the behavioral results, Chen and colleagues further analyzed the effects of CLP290. After discounting a direct effect on lumbosacral function, they explored the possibility that the drug acted by countering a process that interferes with the formation of a neuronal bridge. They hypothesized that the dual hemisections led to a downregulation of KCC2 and a consequent state of over-excitation that disrupts adaptive plasticity. Supporting this, they showed that increasing KCC2 expression in the intervening tissue also promoted the recovery of locomotor function. [61]. Immunohistochemistry revealed that a marker of cellular activity (c-Fos) was highly expressed in the dorsal central grey in the region between the dual hemisections. Treatment with CLP290 or upregulating KCC2 expression attenuated this over-activity and promoted neuronal activity in the intervening intermediate laminae. Using electrophysiology, they showed that re-establishing GABAergic inhibition, by increasing KCC2 expression, augmented the electrical response evoked in a hindlimb muscle (tibialis anterior) in response to cortical stimulation, demonstrating that the treatment promoted the formation of a neuronal relay [61]. The results suggest that over-excitation secondary to ionic plasticity interferes with motor recovery after SCI and that restoring KCC2 functionality with CLP290 or other means can provide a meaningful therapeutic option to improve motor function.

### 3.5. Summary

Considerable evidence now exists that SCI reduces the expression of KCC2, which decreases the inhibitory effect of GABA. Removing this brake on neural activity allows a state of over-excitation to develop within the ventral spinal cord that drives spasticity. New data demonstrate that over-excitation can also interfere with the adaptive rewiring of spinal circuits. Treatments that target ionic plasticity, to promote KCC2 function (CLP290) or attenuate the inward flow of Cl^−^ (bumetanide), have been shown to provide therapeutic benefit in preclinical studies. Importantly, animal research suggests that exercise alleviates spasticity by up-regulating KCC2, providing a mechanistic explanation for the beneficial effects of physical therapy in humans. Further work is needed to determine whether pharmacologically targeting ionic plasticity brings added benefit in clinical treatment, especially for those with injuries that preclude exercise.

## 4. Pain

SCI fosters spasticity by enabling a state of neural over-excitation within the ventral (motor) region of the spinal cord. Injury has a parallel effect on neural excitability in the dorsal region, which enables the sensitization of neural circuits that underlie the processing of pain (nociceptive) signals, broadening the range of stimuli that can engage nociceptive systems and amplifying both the elicited behavioral response and the signal relayed to the brain [19,62]. As we describe below, the loss of GABA-dependent inhibition fuels the development of nociceptive sensitization, which has been linked to the emergence of chronic pain [4,63].

### 4.1. Kinds of Pain

Pain is defined as an unpleasant feeling in the body and can range vastly in severity, duration, quality, and cause [64]. In general, pain is a necessary and important signifier of potentially damaging stimuli. It results from injury or inflammation and indicates physical harm. There are, however, numerous instances where pain is maladaptive—pain that is not tied to acute damage to the body or outlasts the damage and is chronic [65]. These cases are detrimental to the individual as well as society, as they lead to decreased quality of life, impaired productivity, and significant medical and societal costs.

Neuropathic pain is a specific category of pain that results from either injury or disease of the peripheral or central neurons of the somatosensory nervous system [66]. It is the result of a variety of insults: diseases such as multiple sclerosis and multiple myeloma; injuries to extremities or the back; infections, such as shingles or syphilis; and various other factors such as long-term heavy alcohol consumption, cancer treatments, vitamin B deficiency, or thyroid problems [67]. It is most often chronic and can be progressive. Neuropathic pain is thought to affect between 6.9 and 10% of the population [66]. Often described as a burning sensation, neuropathic pain is spontaneous and can be excruciating. Patients can experience the feeling of pins and needles, altered temperature sensations, numbness, or some combination of these symptoms [68]. Neuropathic pain is typically accompanied by hyperalgesia (exaggerated pain sensations to painful stimuli) and allodynia (pain sensations to innocuous stimuli).

There are currently few treatment options for neuropathic pain and prognosis is poor. Traditional analgesics are often not effective. Over-the-counter analgesics do not affect the neural activity that drives the pain and while prescription opiates may temporarily alleviate the symptoms, they come with drastic side effects and significant addictive potential that limit their use. Other classes of drugs used to treat neuropathic pain include anticonvulsants and anti-depressants, both of which are plagued with spotty efficacy and significant side effect profiles [68]. As a result of the poor therapeutic success, patients with neuropathic pain often experience constant pain that leads to decreased quality of life, depression, and even suicide.

Maladaptive pain can develop from numerous inciting injuries but it shares the common feature of altered levels of neural activity [67]. These changes can occur at the peripheral, spinal, or supraspinal levels. Imbalances between excitatory and inhibitory signaling alter the modulation of pain signals and are implicated in both acute and chronic maladaptive pain. This suggests a link to ionic plasticity, and indeed, there is now a significant body of research suggesting just that.

### 4.2. Pain after Spinal Cord Injury

#### 4.2.1. Noxious Stimulation Sensitizes Nociceptive Circuits in the Spinal Cord

Our experience of pain is modulated by both peripheral and central processes. At the peripheral level, the cellular environment in the region of injury and processes within the dorsal root ganglia (DRG) can modulate neural activity related to tissue damage. Sensory neurons are formed from a type of bipolar cell (pseudo-unipolar), with the cell body in the DRG and (axonal) projections to both the periphery and spinal cord [69]. Interestingly, these cells have a high intracellular concentration of Cl^−^ attributable to their expression of NKCC1, but not KCC2. As a consequence, engaging GABAergic neurons that presynaptically innervate a sensory neuron after it enters the spinal cord does not cause an influx of Cl^−^ (Sorkin et al., 2018). At moderate levels, opening some GABA-A channels will have a shunting effect that disrupts the propagation of an electrical impulse. At higher levels of activity, engaging the GABA-A receptor will have a depolarizing effect [primary afferent depolarization (PAD)], which can produce antidromic activity in the sensory neuron (dorsal root reflex [DRR]). Antidromic activity in unmyelinated C fibers can trigger the peripheral release of substance P, calcitonin gene-related peptide (CGRP), and pro-inflammatory cytokines, producing warmth, redness, and pain to touch in the innervated region. These excitatory effects are blocked by intrathecal application of the GABA-A antagonist bicuculline [69].

Unmyelinated (C fibers) and myelinated (A delta) afferent neurons that conduct nociceptive signals project to lamina II (substantia gelatinosa) and laminae I & V of the spinal cord, respectively [70,71]. At this stage, nociceptive activity is regulated by both local processes and descending fibers. Of particular relevance to the present review, prolonged barrages of C fiber activity can sensitize nociceptive circuits within the dorsal horn, a phenomenon that was historically known as central sensitization [72,73]. This enhanced neural activity can amplify locally organized behavioral responses (spinal reflexes) and the nociceptive signal relayed to the brain. It can also transform how non-noxious stimuli (e.g., touch) are processed, enabling myelinated A beta fibers to drive a pain-like response [72]. This transformation is thought to underlie the phenomenon of allodynia. Interestingly, the induction of central sensitization can bring about a lasting modification in how nociceptive signals are processed, laying down a kind of memory that can fuel chronic pain [74,75]. This plasticity is mediated by cellular processes analogous to those identified in brain regions linked to learning and memory, such as the hippocampus. Of note, nociceptive sensitization depends upon a form of N-methyl-D-aspartate (NMDA) receptor-mediated plasticity.

Afferent nociceptive signals are then relayed to the brain, where higher-level processes can modulate psychological pain. In addition, brain mechanisms can regulate incoming nociceptive signaling through descending fibers [76,77]. For example, descending serotonergic fibers can quell nociceptive activity within the dorsal horn. This process, which has been linked to the activation of the 5HT-1A receptor [57,78], helps to maintain a homeostatic balance within the spinal cord that counters prolonged bouts of over-excitation. As we will see below, the loss of these descending fibers after SCI can enable nociceptive sensitization and does so, in part, because it engages a transformation within the dorsal horn that lessens GABAergic inhibition.

To study nociceptive sensitization, researchers engage afferent nociceptive fibers using electrical stimulation or the application of an irritant (e.g., capsaicin, the active ingredient of chili peppers). Both treatments have been shown to induce a modification within the dorsal horn that amplifies the electrophysiological response, reactivity to mechanical stimulation, and afferent signaling to the brain [72,73]. At a cellular level, nociceptive sensitization is evident from markers of neural activity [e.g., cFos and the phosphorylation of ERK (pERK)] [79]. Interestingly, disconnecting the brain from the lower (lumbosacral) spinal cord using a rostral thoracic (T2) transection amplifies cellular indices of nociceptive sensitization [80]. This is consistent with other work demonstrating that descending fibers quell electrophysiological correlates of nociceptive sensitization and the development of spinally-mediated long-term potentiation (LTP) [77]. This quieting effect has been linked to serotonergic pathways that descend through the dorsal lateral funiculus [81,82].

Studies in our laboratory have examined whether neurons in the spinal cord can support simple forms of learning in the absence of brain input [83]. To explore this issue, rats received a rostral spinal cord transection, and the capacity to learn was evaluated using stimuli applied to the tail and/or hindlimbs. In the course of these studies, we discovered that exposure to intermittent electrical stimulation, at an intensity that engages C fibers, induces a form of maladaptive plasticity that interferes with learning about response-outcome relations [84,85]. The development of this effect was accompanied by increased reactivity to mechanical stimuli applied to the hind paws, an allodynic-like effect indicative of nociceptive sensitization [86]. Given this observation, we tested whether other treatments that induce central sensitization (e.g., application of capsaicin, formalin, or carrageenan to one hind paw) impair adaptive learning. We found that they did [86,87]. We further showed that the development of maladaptive plasticity is blocked by pretreatment with an NMDA receptor (NMDAR) antagonist, leading us to suggest that noxious stimulation impairs adaptive learning because it saturates NMDA receptor-mediated plasticity [86].

#### 4.2.2. Ionic Plasticity Fosters Nociceptive Sensitization after SCI

There was, however, another finding that appeared to challenge the link between maladaptive plasticity and nociceptive sensitization. There is considerable evidence that intrathecal (i.t.) application of a GABA-A antagonist (e.g., bicuculline) enhances nociceptive processing within the spinal cord, producing a state that increases behavioral reactivity to noxious and non-noxious stimulation [88,89]. This is, of course, consistent with the idea that GABAergic interneurons normally have an inhibitory effect on nociceptive processing within the spinal cord. Blocking this tonic effect with bicuculline removes this brake, inducing a state akin to nociceptive sensitization. Given these observations, we naturally hypothesized that pretreatment with bicuculline would, if anything, amplify nociceptive sensitization and the development of maladaptive plasticity. The opposite was observed—pretreatment with bicuculline blocked the development of the learning impairment [90]. Subsequent work revealed that the enhanced mechanical reactivity observed in spinally transected rats exposed to noxious electrical stimulation is also blocked by bicuculline [91]. The implication is that the release of GABA, and the activation of the GABA-A receptor, play an essential role in the development of maladaptive plasticity. The results also suggest that pretreatment with bicuculline should block other instances of nociceptive sensitization. Supporting this, we showed that pretreatment with bicuculline prevents the enhanced mechanical reactivity elicited by acute inflammation [induced by intrathecal application of the endotoxin lipopolysaccharide (LPS)] or the application of an irritant (capsaicin) to one hind paw [91]. Importantly, bicuculline also blocked the expression of cellular indices of nociceptive sensitization (*c-fos* and pERK).

Our results suggest that after SCI, the release of GABA within the spinal cord may drive rather than inhibit the development of nociceptive sensitization. We recognized that this shift could reflect the consequences of ionic plasticity, wherein a loss of GABAergic inhibition enables over-excitation in the dorsal horn just as it does in the ventral spinal cord. To explore this possibility, we examined whether a spinal transection induces a shift in membrane-bound KCC2 within the dorsal horn. We found that KCC2 was downregulated within 24 h of injury [91], which would lead to a rise in intracellular Cl^−^ and a loss of GABAergic inhibition. If this process plays a key role, blocking the inward flow of Cl^−^ through the NKCC1 channel with bumetanide should restore GABAergic inhibition and reverse how bicuculline affects capsaicin-induced sensitization in transected rats. As predicted, after i.t. bumetanide, bicuculline no longer had its paradoxical antinociceptive effect [91]. Instead, bicuculline fostered the development of enhanced mechanical reactivity in capsaicin-treated rats, an outcome that parallels what is observed in uninjured rats. The converse prediction is that blocking the KCC2 channel with DIOA in uninjured rats should reverse how bicuculline affects nociceptive sensitization, in this case eliminating its pro-nociceptive effect and unveiling a bicuculline-induced antinociception analogous to what is observed after SCI. The results were as predicted [91].

We and others have shown that BDNF enables adaptive plasticity after SCI [92,93,94,95]. BDNF also has a protective/restorative effect that counters the development of the learning impairment induced by intermittent electrical stimulation [96]. Integrating these observations with the studies described above suggests that BDNF may counter the emergence of nociceptive sensitization after SCI. As predicted, we found that BDNF blocked the development of enhanced mechanical reactivity after capsaicin treatment in spinally transected rats [80]. The drug also blocked the expression of a cellular marker of nociceptive sensitization (pERK) and increased the expression of membrane-bound KCC2. Interestingly, BDNF has the opposite effect in uninjured animals [80], inducing an allodynic-like increase in mechanical reactivity and downregulating membrane-bound KCC2. The latter observations are consistent with the data described below and imply that SCI transforms how BDNF affects KCC2. As noted above (see 2.3.2), this transformation may be linked to two effectors of TrkB signaling: Shc and PLCγ. When both are expressed, BDNF appears to downregulate KCC2 [5]. When PLCγ is absent, as occurs early in development and after SCI [33,42], BDNF has the opposite effect.

As noted above, earlier work showed that serotonergic fibers that descend through the DLF can quell neural excitability within the dorsal horn [77,78], a homeostatic effect that limits spinal cord plasticity and the development of nociceptive sensitization. Given this, and other data implicating descending fibers in the regulation of KCC2 [4,91], we hypothesized that the downregulation of KCC2 after SCI is linked to a loss of serotonergic fibers within the DLF. Supporting this, we showed that lesions limited to the DLF had the same effect as a complete transection, producing a downregulation in KCC2 [97]. DLF lesions also reversed how bicuculline affects capsaicin-induced enhanced mechanical reactivity, causing the drug to have an antinociceptive effect analogous to that observed after spinal transection. A parallel pattern of results was obtained when uninjured animals received the 5HT-1A antagonist WAY-100635 before capsaicin treatment. Conversely, in transected rats, application of the 5HT-1A agonist 8OH-DPAT increased membrane-bound KCC2 and eliminated the antinociceptive effect of bicuculline.

A key question that remains unanswered is whether these spinally-mediated alterations impact pain signaling to the brain. We recognized that this issue could be addressed in animals that had received DLF lesions, which should cause a downregulation of KCC2 in the spinal dorsal horn but not affect ascending pain signals [97]. To evaluate whether spinally-mediated ionic plasticity affects a brain-dependent measure of pain, we used a place conditioning task [98] in which animals are exposed to two distinctive chambers (contexts). Before being placed in one context, animals were given bicuculline followed by the application of capsaicin to one hind paw. Prior to being placed in the other context, animals received a vehicle injection before capsaicin treatment. In sham-operated rats, bicuculline should enhance capsaicin-induced pain, causing animals to develop a greater aversion to that context. To test whether this occurred, rats were given a preference test and as predicted, sham-operated rats avoided the context where they received bicuculline before capsaicin treatment [97]—implying that the GABA antagonist had indeed enhanced psychological pain. Animals that had undergone DLF lesions exhibited the opposite pattern of results, demonstrating a preference for the context where they had received bicuculline before capsaicin treatment, which suggests that the GABA-A antagonist had attenuated psychological pain. The results imply that after SCI, GABA amplifies nociceptive sensitization in the spinal cord and enhances pain signaling to the brain.

Collectively the studies reviewed above show that SCI alters GABA function in the dorsal horn, mirroring the induction of ionic plasticity within the ventral region. In both, the change has been related to a loss of descending 5HT fibers. The reduction in KCC2 removes a brake on neural excitability that fosters nociceptive sensitization and psychological pain.

#### 4.2.3. Ionic Plasticity Fosters Chronic Pain after SCI

The studies described above showed that surgically cutting communication with the brain brings a change in GABA function that promotes nociceptive sensitization. Are these observations relevant to the development of chronic pain after a more clinically relevant contusion injury that spares some ascending/descending fibers? This issue was addressed by Cramer and colleagues. They first tested the effects of bumetanide, an NKCC1 antagonist, on withdrawal latency time, a behavioral indicator of hyperalgesia and neuropathic pain [4]. Mirroring Huang et al., 2016, they found that bumetanide increased the latency to respond, indicating its anti-hyperalgesic effects [4]. To understand the state of Cl^−^ balance in the spinal cord after SCI, Cramer et al. 2008 assessed both NKCC1 and KCC2 protein levels. They found that fourteen days after SCI, NKCC1 is elevated at the injury epicenter, whereas KCC2 is decreased [4]. Rostral samples did not show significant changes in NKCC1 or KCC2 expression. Testing 21–35 days after SCI revealed that NKCC1 was reduced along with KCC2 [4]. What is of particular interest is that these chronic changes in protein expression were only present in rats with thermal hyperalgesia [4]. Furthermore, changes in protein levels related to ionic plasticity paralleled behavioral measures of neuropathic pain. This provides strong evidence that maladaptive pain is mechanistically linked to ionic plasticity.

#### 4.2.4. Restoring GABAergic Inhibition Attenuates Pain after SCI

Given the role of ionic plasticity in the development of pain after SCI, it follows that restoration of GABAergic inhibition should provide therapeutic relief. Sánchez-Brualla and colleagues addressed potential therapeutic options when examining the role of serotonergic signaling in neuropathic pain [99]. They tested the analgesic effect of a 5HT-2A receptor agonist, (4-bromo-3,6-dimethoxybenzocyclobuten-1-yl) methylamine hydrobromide (TCB-2), in neuropathic pain following SCI [99]. After establishing thermal and mechanical allodynia in SCI, Sánchez-Brualla, et al. 2018 tested the effects of TCB-2. They found that both thermal and mechanical allodynia were alleviated by treatment. Relief was more robust in mechanical allodynia [99]. To test the mechanism of TCB-2′s antinociceptive effects, they then co-administered the KCC2 inhibitor DIOA. DIOA coadministration prevented the TCB-2-induced antinociceptive effects, demonstrating that they are KCC2-dependent [99]. Sánchez-Brualla and colleagues then confirmed the role of KCC2, demonstrating that TCB-2 administration significantly increased KCC2 immunostaining in lamina I and II neuronal membranes [99].

The therapeutic success was extended to human patients by Zarepour and colleagues who evaluated whether bumetanide alleviates neuropathic pain after SCI [100]. They performed a pilot trial on 14 SCI patients and assessed pain scores as well as the expression of NKCC1 and KCC2 after 19 weeks of treatment. Bumetanide decreased pain scores on several pain scales [100]. This therapeutic improvement was accompanied by increased KCC2 protein expression suggesting bumetanide drove improved GABAergic inhibition and that the shift in GABAergic inhibition improved pain symptoms [100].

Collectively, the results above demonstrate that ionic plasticity after SCI drives nociceptive sensitization and fosters chronic pain. Ionic plasticity after SCI also causes overexcitation that inhibits motor function. The results provide hope demonstrating that restoring Cl^−^ concentrations after SCI will bring therapeutic successes. The findings also highlight a conceptual shift. Our past work focused on active processes that could drive over-excitation, such as NMDA receptor-mediated plasticity and the trafficking of Ca^++^ permeable α-amino-3-hydroxy-5-methyl-4-isoxazolepropionic acid (AMPA) receptors to the active zone of the synapse [86,101,102,103]. What we now recognize is that over-excitation may emerge not because the gas pedal has been pushed to the floor, but rather, because an inhibitory brake has been removed. This shift parallels the evolution in thinking regarding the development of other forms of pathological over-excitation, reviewed below.

### 4.3. Ionic Plasticity Can Fuel Pain in the Absence of Spinal Cord Injury

SCI can enable a modification within the caudal spinal cord that attenuates GABAergic inhibition, and this instance of ionic plasticity fosters the sensitization of nociceptive circuits within the dorsal horn. Are these effects limited to instances of pain after SCI or does this work have more general implications? New findings support the latter, providing evidence that other processes (e.g., inflammation, peripheral injury) can promote pain through ionic plasticity in the absence of SCI.

#### 4.3.1. Acute Pain

Nomura and colleagues explored whether KCC2 and NKCC1 play a role in the development of enhanced pain in uninjured animals using the formalin model, wherein the irritant is microinjected into one hind paw. They found that KCC2 was significantly reduced in the ipsilateral dorsal horn shortly after injection and that levels returned to baseline within an hour [104]. KCC2 mRNA levels were unchanged, suggesting the altered KCC2 was the result of a change in protein trafficking rather than synthesis. In contrast to others, Nomura et al. 2006 did not find any changes in NKCC1 expression in the dorsal horn following formalin injection [104]. These results suggest that acute pain can bring about a rapid decline in KCC2.

Tsuruga and colleagues examined the mechanisms that mediate the downregulation of KCC2 in response to acute pain [105]. Given that past studies implicated BDNF-TrkB signaling, they posited that this signal pathway plays a pivotal role. Tsuruga et al. 2016 first confirmed peripheral treatment with formalin reduces KCC2 expression in the dorsal horn [105]. They then showed that disrupting TrkB signaling with K252a prevents the downregulation of KCC2 [105].

Funk and colleagues examined potential drivers of ionic plasticity in inflammatory hyperalgesia. To address this issue, they evaluated the effect of several inflammatory mediators on Cl^−^ homeostasis in DRG cells [106]. Cells were bathed in a combination of NGF, ATP, bradykinin, and PGE2 for several hours and then analyzed. Funk and colleagues found that inflammatory mediators increased intracellular Cl^−^, caused an early increase in NKCC1 phosphorylation, and later led to decreased KCC2 expression and increased NKCC1 expression [106]. The results suggest that inflammatory mediators can drive ionic plasticity.

Taken together, the findings show that ionic plasticity plays a role in acute pain. What is clinically more important is whether this process contributes to the development of chronic pain.

#### 4.3.2. Neuropathic Pain

Coull et al. (2003) examined the state of anion balance in spinal lamina I neurons, part of the main spinal pain pathway, in neuropathic pain. Given that chronic pain states are thought to result, at least in part, from disinhibition of activity in the dorsal horn, they sought to identify the mechanisms that underlie this effect [107]. Coull et al., 2003 found a trans-synaptic reduction in KCC2 following a peripheral nerve injury that disrupts Cl^−^ homeostasis in lamina I neurons of the dorsal horn [107]. They discovered that the shift in the anion concentrations drove normally inhibitory currents to become excitatory, increasing the general state of excitability. To confirm that these effects were clinically relevant, Coull and colleagues blocked KCC2 function and knocked down KCC2 levels. Both manipulations decreased the nociceptive thresholds [107]. These results provide behavioral evidence that decreased KCC2 activity is sufficient to drive neuropathic pain similar to that after peripheral nerve injury.

Coull and colleagues went on to explore the mechanisms that underlie the downregulation of KCC2 in neuropathic pain. They found that tactile allodynia and the depolarizing shift in anions of lamina I neurons after injury require endogenous BDNF [108]. This BDNF comes from the microglia within the spinal cord. GABA changed from inhibitory to excitatory when rats were treated with ATP-stimulated microglia [108]. The changes in paw withdrawal and GABA functionality caused by the ATP-stimulated microglia are dependent upon BDNF-TrkB signaling. Delivering BDNF locally to the lamina I neurons of the dorsal horn led to a decrease in paw withdrawal threshold [108]. With these results, Coull and colleagues provided the first concrete evidence that ionic plasticity is important in neuropathic pain.

The mechanisms driving KCC2 expression in neuropathic pain were further detailed by Miletic et al., 2008. Following loose ligation of the sciatic nerve, they first demonstrated that there is decreased KCC2 in the ipsilateral dorsal horn [109]. They were able to prevent this decrease by pretreating animals with K252a, a tyrosine kinase receptor blocker, or with TrkB/Fc, a chimera protein that sequesters BDNF. Additionally, these treatments blocked behavioral measures of enhanced pain [109]. The fact that KCC2 expression was preserved with K252a and TrkB/Fc implies that the downregulation of KCC2 in this model is the result of activation of the BDNF-TrKB signaling cascade. Supporting this, Miletic and colleagues showed that BDNF is upregulated after nerve ligation [109]. Finally, they demonstrated that the downregulation of KCC2 was transient and returned to baseline levels seven days after nerve ligation, even though pain behaviors were still present [109]. Miletic et al. concluded that these ionic plasticity changes are responsible for increased neural excitability and early pain behaviors after injury. Their results paralleled Tsuruga’s findings in acute pain, suggesting that in both acute pain and neuropathic pain, KCC2 downregulation is driven by BDNF.

Galan and colleagues hypothesized that an increase in functional NKCC1 in the dorsal horn may contribute to some hyperalgesic states [110]. They evaluated visceral pain, by applying intracolonic capsaicin. After capsaicin treatment, NKCC1 was increased in the membrane-bound fraction and decreased in the cytoplasmic fraction of lumbosacral spinal cord homogenates. Interestingly, this shift in NKCC1 was present only in the region of the spinal cord that receives afferent input from the colon [110]. Rapid phosphorylation of NKCC1 was also observed following intracolonic capsaicin [110]. These changes would increase NKCC1 functionality and bring a transient shift in Cl^−^ transport that would drive depolarizing GABA-A currents, increasing excitability and enhancing pain.

Pitcher and colleagues further explored the role of Cl^−^ homeostasis, and NKCC1 in neuropathic pain using electrophysiology. They first demonstrated that high-threshold nociceptive neurons have lower activation thresholds after exposure to capsaicin and exhibit greater activity [111]. The activity of wide dynamic range neurons also increased. To assess whether these changes were related to an alteration in intracellular Cl^−^, Pitcher et al., 2010 applied bumetanide [111]. In both wide dynamic range and nociceptor-specific neurons that demonstrated signs of sensitization, bumetanide returned activity and thresholds to baseline [111]. These results demonstrate that blocking NKCC1, and thus restoring GABAergic inhibition, reverses capsaicin-induced sensitization. Together, the above results show that neuropathic pain secondary to peripheral injury is, at least in part, linked to ionic plasticity.

#### 4.3.3. Restoring GABAergic Inhibition Attenuates Pain

The mounting evidence that a shift in intracellular Cl^−^ concentrations sets the stage for enhanced pain has led researchers to explore the therapeutic potential of treatments that target ionic plasticity. Yuan and colleagues examined ionic plasticity in knee osteoarthritis. After inducing knee osteoarthritis, a treatment regimen of electroacupuncture (EA) was administered [112]. Rats that received EA showed decreased mechanical allodynia. To explore the underlying pathway Yuan and colleagues measured 5HT-2A expression. EA increased the expression of 5HT-2A in the dorsal horn [112]. To confirm a causal link, Yuan et al. administered Ketanserin, a 5HT-2A antagonist. Ketanserin reversed the beneficial effect of EA on mechanical allodynia [112]. Furthermore, administration of the 5HT-2A receptor agonist DOI mimicked the beneficial effects of EA. To examine what downstream pathway is affected by EA. Yuan and colleagues assessed GABA-A and KCC2 expression. Both were increased following EA treatment and these effects were mirrored by DOI administration and blocked by Ketanserin administration [112]. Knowing the signaling cascade linking KCC2 and 5HT-2A, Yaun and colleagues administered the PLC inhibitor U73122. Intrathecal application of U73122 reversed the behavioral benefit of decreased mechanical allodynia seen after EA as well as the EA-induced increase in KCC2 expression [112]. Collectively, these results demonstrated that electroacupuncture can be used to treat chronic osteoarthritis pain and that the success is the result of activation of the 5HT-2A-Gq-PLC pathway and resultant KCC2 upregulation.

Zhao and colleagues investigated the BDNF-TrkB pathway and the role of ionic plasticity using a brachial plexus avulsion model [113]. Disrupting BDNF-TrkB signaling with K252a reduced both mechanical hyperalgesia and cold allodynia. As expected, BDNF and TrkB were elevated after brachial plexus avulsion and KCC2 levels were decreased [113]. K252a administration successfully restored BDNF, TrkB, and KCC2 levels to those of the sham group [113].

Further evidence that targeting ionic plasticity has therapeutic potential in the treatment of neuropathic pain was provided by Yeo and colleagues [114]. A drug screen identified kenpaullone, a cancer therapeutic, that augments KCC2. Using a peripheral nerve constriction model of neuropathic pain model, they showed kenpaullone attenuates behavioral signs of pain in vivo [114]. To confirm that the effect on pain was secondary to its effect on KCC2, Yeo and colleagues co-administered VU0240551, a KCC2 inhibitor. As expected, the antinociceptive effect of kenpaullone was eliminated. They also found that KCC2 expression is restored by kenaullone administration [114]. Furthermore, kenpallone attenuated neuropathic pain in a bone cancer pain model [114].

Building on their work demonstrating that a 5HT-2A agonist (TCB-2) attenuates chronic pain after SCI, Sánchez-Brualla and colleagues examined whether this drug would counter neuropathic pain after spared nerve injury [99]. TCB-2 administration did not alleviate allodynia in this pain model. However, TCB-2 administration did restore membrane-bound KCC2 expression [99]. These results demonstrate that KCC2 restoration secondary to 5HT-2A signaling is not sufficient for antinociception following spared nerve injury, but it is following SCI. These divergent results imply injury-specific complexities in the role of 5HT signaling and ionic plasticity in neuropathic pain.

#### 4.3.4. Role of Sex in Pain

A key question in the development of effective therapeutics is whether the mechanisms that underlie pain vary with sex. While the behavioral features of neuropathic pain are no different between the sexes, there are underlying sex differences in the neuroimmune signaling that drives the phenomenon [115]. With the hopes of identifying a unifying mechanism, Mapplebeck et al., 2019 examined whether the role of ionic plasticity in neuropathic pain is sex-specific. They first confirmed that synaptic inhibition in the dorsal horn is dependent on KCC2 in both sexes. They did so by blocking KCC2 in uninjured rats [116]. Mapplebeck et al., 2019 went on to confirm that KCC2 is downregulated in both males and females after spinal nerve ligation [116]. They then tested whether this KCC2 downregulation was responsible for the allodynia seen after injury. They used CLP290, a KCC2 enhancer, to reverse KCC2 downregulation and found that this had anti-allodynic effects in both males and females [116]. Thus, after spinal nerve injury, Cl^−^ dysregulation mediates the consequent allodynia in both sexes.

Mapplebeck and colleagues also showed that KCC2 downregulation was sufficient to induce neuropathic pain in uninjured mice. KCC2 was inhibited using DIOA or downregulated with BDNF. Both treatments reduced behavioral and electrophysiological measures of neuropathic pain in males and females [116]. The opposite is also true. Correcting Cl^−^ dysregulation after injury reverses behavioral and electrophysiological signs of neuropathic pain in both sexes [116].

Other recent data suggest that sex differences may exist in a model of inflammatory pain [117]. Application of the irritant Freund’s adjuvant to a rat’s hind paw induced mechanical allodynia in both male and female rats. The development of enhanced pain was linked to BDNF-TrkB signaling, and a downregulation of KCC2, in male but not female animals. Interestingly, Freund’s downregulated KCC2 through a BDNF-dependent process in females ovariectomized prior to maturity. Ex vivo assessment of tissue from human donors also revealed sex differences in KCC2 expression and BDNF signaling [117]. Clearly, further work is needed to determine how sex and hormones influence the processes engaged in pathological pain.

#### 4.3.5. Opiate Hyperalgesia

A further complicating factor in pain management stems from the fact that treatments used to alleviate pain can, in of themselves, impact Cl^−^ homeostasis. Opiates are of particular concern because these drugs are widely used to treat pain and because repeated administration can engage a hyperalgesic state that causes a paradoxical decrease in pain threshold. Ferrini et al., 2013 confirmed that animals that were repeatedly given a high dose of the opiate morphine exhibit hyperalgesia, which they related to altered Cl^−^ extrusion in lamina I neurons of the spinal cord [118]. They went on to show morphine treatment induces a decrease in KCC2 immunoreactivity in lamina I [118]. These results suggest that morphine impairs Cl^−^ homeostasis by reducing KCC2 activity. To further explore the underlying mechanisms, they targeted microglial activity and BDNF signaling. Depletion of microglia prevented morphine-induced allodynia, as did administration of anti-TrkB antibody [118]. These results demonstrate that the development of morphine-induced hyperalgesia is dependent upon microglial BDNF driving ionic plasticity.

Given the link between morphine-induced hyperalgesia, changes in Cl^−^ extrusion, and molecular pathways that parallel those in neuropathic pain, Ferrini and colleagues tested whether morphine-induced hyperalgesia can be resolved by restoring the Cl^−^ balance [119]. The KCC2 enhancers CLP257 and CLP290 prevented morphine-induced hyperalgesia [119]. These results suggest that treatments that restore Cl^−^ homeostasis may prove a useful adjunct in pain management. Further work is needed to determine whether ionic plasticity also contributes to the adverse effect that opiate treatment has on tissue sparing and long-term recovery after SCI [120,121].

#### 4.3.6. Diabetic Neuropathy

One of the most common complications of diabetes mellitus is nerve damage (diabetic neuropathy), which can fuel chronic pain. Like other forms of maladaptive pain, diabetic neuropathy is associated with spontaneous pain, allodynia, and hyperalgesia and it is mechanistically linked to hyperexcitability and increased activity of the neurons involved [122]. Morgado and colleagues sought to address the role of decreased inhibition in diabetic neuropathy. In their model paradigm, diabetes was induced with streptozotocin, which produced mechanical and thermal hyperalgesia [123]. They found increased GABA and a significant decrease in KCC2 expression in the dorsal horn of symptomatic diabetic rats relative to controls [123]. These changes would flip GABA from inhibitory to excitatory, which would drive neuropathy and pain.

Jolivalt et al., 2008 also examined the state of ionic plasticity as it relates to diabetic neuropathy. They confirmed that diabetes decreases KCC2 levels in the spinal cord and found that it does not affect NKCC1 or GABA-A receptor expression [124]. To explore how these diabetes-induced changes in GABA function affect pain processing, diabetic rats had the irritant formalin applied to one hind paw, which induced flinching, tactile allodynia, and thermal hyperalgesia. Administration of the GABA-A antagonist bicuculline dose-dependently attenuated formalin-induced flinching and allodynia. These results parallel those obtained by Huang [91], who showed that bicuculline attenuates capsaicin-induced allodynia and pain after SCI. Interestingly, bicuculline did not rescue thermal hyperalgesia in diabetic rats [124]. The overall pattern of results suggests that diabetes transforms how GABA affects neural excitability, causing it to have an excitatory effect that drives nociceptive sensitization. Supporting this, diabetic animals did not exhibit RDD of the spinal H-reflex, implying a loss of GABAergic inhibition. Furthermore, pharmacologically blocking KCC2 in the spinal cord with DIOA in non-diabetic rats yields a diabetic-like phenotype, with a loss of RDD and reversal in how bicuculline affects formalin-induced allodynia [124]. Again, this parallels the results reported by Huang [91].

### 4.4. Summary

Research has shown that ionic plasticity plays a key role in the development of pain after SCI, peripherally induced neuropathic pain, and other non-neuropathic forms of both acute and chronic pain. The variety of insults that can drive this process converges with a shift in Cl^−^ homeostasis. Hasbargen pointed out the distinct ways that NKCC1 and KCC2 expression levels are altered [63]. That is, with some forms of pain NKCC1 levels alone are altered, whereas other pain-inducing stimuli alter KCC2 levels only, and still other forms of pain alter both proteins. Whether NKCC1 expression is increased, KCC2 expression is decreased, or both, high intracellular Cl^−^ concentrations result. At a minimum, this lessens a brake on nociceptive circuits within the dorsal horn. High local concentrations of Cl^−^ can go further, enabling an efflux of the anion that depolarizes the post-synaptic cell [125]. The immediate consequence is the amplification of pain signals to the brain. Beyond this, heightened neural activity can foster the rewiring of neural circuits in the dorsal horn, enabling non-noxious stimuli to engage nociceptive pathways and laying down a kind of memory that can drive chronic pain [74,75].

Given the role of ionic plasticity in central sensitization and the development of pain after SCI along with its identification in numerous other forms of pain, targeting the affected pathway provides therapeutic promise across numerous settings. The described research demonstrates a mechanistic commonality that may yield an entirely new line of treatments for neuropathic pain and pain in general.

## 5. Contributions of Ionic Plasticity to Brain-Mediated Pathophysiology

There is ample evidence that ionic plasticity impacts neural function within the spinal cord, driving spasticity and pain in response to injury and inflammation. The key feature is aberrant neural activity linked to a loss of GABA-dependent inhibition. Do these observations have implications that extend beyond the spinal cord, to inform our understanding of pathology within the brain? In the material that follows, we will see that ionic plasticity does indeed play a role in a number of brain-mediated diseases, including seizures, epilepsy, developmental disorders, and addiction.

### 5.1. Seizures and Epilepsy

When considering an imbalance of excitation and inhibition in the brain one first thinks of the family of disorders characterized by over-excitation of the brain itself: seizures, and epilepsy. A seizure is a transient increase in the electrical activity of the brain [126]. Seizures may be induced by countless factors including infections, injuries, metabolic alterations, genetic mutations, or medications. If seizures arise spontaneously, they are deemed epileptic. Epilepsy is characterized by recurrent, unprovoked seizures [126].

Epilepsy is a multi-factorial family of diseases and can be divided into various categories based on seizure type, onset, brain region affected, and other factors. Epilepsy is the most common serious brain disorder worldwide, affecting approximately 50 million people [127]. Epilepsy is costly in many ways—it is associated with cognitive impairment, psychosocial dysfunction, increased mortality rates, and over $12 billion annually to the United States economy alone [128]. Given the current therapeutic options for seizures, one-third to one-half of epilepsy patients continue to have seizures despite medication [129].

There is now mounting evidence that seizures, and more specifically epilepsy, are the result of deficient GABA inhibition. Various causal factors have been identified in epilepsy patients and seizure models, such as loss of GABAergic interneurons, downregulation of GABA receptors, and decreased spontaneous GABAergic activity [130]. Impairment of KCC2 activity would predispose one to seizures by interfering with the GABA-A mediated inhibitory tone. This would yield an imbalance of excitatory over inhibitory activity.

#### 5.1.1. Role of Ionic Plasticity in Seizures and Epilepsy

Moore and colleagues explored the role of KCC2, and in turn ionic plasticity, in seizure activity. To do this, they developed a knock-in of KCC2-T906A/T1007A, a double-point alanine substitute mutant line that prohibits the inhibitory phosphorylation of the affected residues, thus increasing KCC2 activity [131]. To prove their model’s efficacy, they assessed Cl^−^ extrusion capacity and found that despite no difference in total or surface KCC2 protein expression levels, mutant mice displayed increased Cl^−^ extrusion [131]. Using 4-aminopyridine, a commonly used chemoconvulsant, they assessed seizure-like activity in vitro. They found that all wild-type slices, but less than half of the mutant slices, exhibited seizure-like activity [131]. Furthermore, in mutant preparations that exhibited seizure-like activity, the onset of activity was delayed, and the percent of time spent in seizure-like activity was reduced. Finally, no mutant slices entered status epilepticus, whereas nearly half of the wildtype preparations did [131]. To confirm that these differences were the result of enhanced KCC2 activity, Moore and colleagues administered VU0463271, a KCC2 antagonist. They found that application of 4-aminopyridine in the presence of VU0463271 resulted in seizure-like activity in all slices, both wildtype and mutant [131]. The onset of and time spent in seizure-like activity were also equalized by the administration of the KCC2 inhibitor. In the presence of VU0463271, both wildtype and mutant slices rapidly entered status epilepticus [131]. Moore and colleagues then confirmed these results using a magnesium deprivation model of seizure induction. The results paralleled those found with 4-aminopyridine, demonstrating that these effects are not seizure-model specific [131].

Moore and colleagues went on to assess the effects of KCC2 enhancement in vivo using the chemoconvulsant kainate. KCC2 phosphorylation at T1007 was increased in wild-type mice one hour after kainate administration [132]. This would decrease KCC2 activity and suggests that seizures drive changes that predispose one to more seizures. Again, the mutant mice demonstrated a delay in seizure onset [132]. Additionally, the mortality rate was significantly reduced in mutant mice, and for those that did die, time of death was significantly delayed compared to wild-type mice [132]. Together, these data demonstrate that preventing the inhibitory phosphorylation of KCC2 at threonines 906 and 1007 enhances KCC2 activity. Furthermore, the resultant increase in activity is sufficient to delay the onset of, and decrease the severity and presence of, chemoconvulsant-induced seizures in vivo.

McNamara and colleagues examined the mechanisms that drive changes in KCC2 in epilepsy. Across several animal models, they found that BDNF was consistently upregulated following seizure activity [133]. They also found that TrkB activation was greater following seizures [133]. While they did not directly correlate this with KCC2 or ionic plasticity, BDNF is known to downregulate KCC2 in the absence of neural injury. Moreover, knocking down TrkB prevented seizure development in the presence of chemoconvulsants. Transient inhibition of TrkB, a conditional knockout of TrkB, or uncoupling of the PLCγ arm from TrkB, all yielded similar results [133]. All of these treatments would prevent BDNF-driven downregulation of KCC2 and all decrease the development of epilepsy. Elimination of one BDNF allele also inhibited seizure kindling [133]. These results strongly suggest that BDNF and TrkB signaling contribute to seizure activity and epileptogenesis, likely through their effects on KCC2.

Further research in humans has demonstrated KCC2 deficiencies and various KCC2 mutations in patients with known epilepsy. In fact, fourteen different mutations of SLC12A5, the gene that encodes KCC2, have been identified in human epilepsy patients thus far [134]. These mutations vary in their effects, but all yield decreased KCC2 function in one way or another. For example, Kahle and colleagues identified two missense variants of KCC2 in generalized epilepsy patients [134]. One led to decreased cell surface expression while the other decreased intrinsic transporter activity. Both mutants also demonstrated decreased phosphorylation on serine 940, which is known to increase KCC2 activity [134].

A specific form of epilepsy, epilepsy of infancy with migrating focal seizures, provides strong evidence that KCC2 dysfunction secondary to genetic mutations leads to seizures. Nine specific KCC2 mutations have been identified in association with epilepsy of infancy with migrating focal seizures [135]. All of the identified mutations result in diminished Cl^−^ extrusion and reduced cell surface expression [135]. Together, this genetic evidence demonstrates the importance of KCC2 and Cl^−^ homeostasis in maintaining normal neuronal excitability and protecting against over-excitation and seizures.

Interestingly, epileptogenic brain tumors also exhibit ionic plasticity and the resultant shift in GABAergic signaling [134]. When Conti et al., 2011, analyzed cortical tissue from regions adjacent to tumors, they found altered expression and activity of both NKCC1 and KCC2 and reduced GABA-driven hyperpolarization [136]. In addition, Pallud and colleagues found a 144% increase in NKCC1 and a 42% decrease in KCC2 expression in patients who had seizures secondary to gliomas [137]. The recurrent thread of ionic plasticity in patients with seizures secondary to a variety of causes suggests a common target for treatment.

#### 5.1.2. Restoring GABAergic Inhibition Attenuates Seizure activity

Re-establishing ionic balance has shown promise in treating epilepsy. Sullivan and colleagues examined the efficacy of CLP290 in a mouse model of neonatal seizures [138]. To assess the therapeutic benefit of CLP290, they tested whether seizures that were resistant to phenobarbital treatment could be “rescued” with the addition of CLP290 [138]. They found that CLP290 significantly reduced the total burden, duration, and frequency of seizures and increased the seizure suppression of phenobarbital. To better understand how CLP290 is affecting KCC2, they tested several KCC2 mutant mice strains. Knock-in phosphorylation at T1007A reduced seizure susceptibility, whereas S940A knock-in increased seizure burden, status epilepticus, and death [138]. Sullivan and colleagues then examined whether the efficacy of CLP290 treatment was mechanistically linked to KCC2 phosphorylation. They found that CLP290 rescued overall KCC2 expression and S940 phosphorylation, both of which lead to increased Cl^−^ extrusion capacity [138]. CLP290 did not significantly affect T1007 phosphorylation. Interestingly, CLP290 administration did not affect TrkB expression suggesting that the beneficial effects of CLP290 seen here are independent of the TrkB pathway [138]. The results identify KCC2 phosphorylation as a mechanism of CLP290 and provide a basis for treating seizures by targeting ionic plasticity.

Clinical work has also shown that restoring GABAergic inhibition can successfully treat epilepsy patients. Eftekhari and colleagues found that NKCC1 inhibition with bumetanide could successfully reduce seizure frequency in adult patients with temporal lobe epilepsy [139]. More recent studies have confirmed bumetanide as a beneficial therapy for temporal lobe epilepsy in adults [140]. There have also been successes in treating pediatric epilepsy [141]. While targeting ionic plasticity has yielded varied results across distinct forms of epilepsy, the role of GABAergic inhibition is undeniably important [134]. Ionic plasticity clearly contributes to the pathogenesis of epilepsy. The changes in NKCC1 and KCC2 likely interact with countless other factors driving the complexity of the family of diseases that constitute epilepsy.

#### 5.1.3. Neurodevelopmental Diseases

Another intriguing line of research implicates ionic plasticity, and the GABAergic switch, in several neurodevelopmental disorders that are associated with increased risk of epilepsy, such as Down syndrome, fragile X syndrome, and Rett syndrome [131]. Deidda et al., 2015 found that the reversal potential of Cl^−^ currents was shifted and GABA signaling was excitatory, rather than inhibitory, in a mouse model of Down syndrome [142]. Furthermore, NKCC1 expression was increased in both the mouse model of Down syndrome and in individuals with diagnosed Down syndrome [142]. Treatment with bumetanide, the NKCC1 inhibitor, restored the Cl^−^ balance and improved hippocampus-dependent memory in adult Down syndrome mice [142]. He and colleagues found that the normal developmental progression of the Cl^−^ reversal potential that drives the switch of GABA activity from excitatory to inhibitory was delayed in a mouse model of fragile X syndrome [143]. This delay was paralleled by increased NKCC1 expression [143].

Duarte et al., 2013 found reduced levels of KCC2, and decreased KCC2/NKCC1 ratios, in the cerebrospinal fluid of Rett syndrome patients [144]. Tang and colleagues further identified a deficit of KCC2 in Rett syndrome neurons that impaired the switch in GABA functionality from excitatory to inhibitory. Additionally, Tang et al., 2016 found that KCC2 is a downstream target of methyl CpG binding protein 2 (MeCP2), which is the single gene mutation known to cause Rett syndrome [145].

The role of ionic plasticity in each of these developmental disorders appears to stem from altered timing or progression of the switch in GABA functionality. The altered Cl^−^ balance drastically affects numerous aspects of neural functioning and neural development. Recognition of this imbalance sheds light on potential therapeutic options, not only for the seizures often associated with these disorders, but also for the cognitive deficits, spasticity, and other symptoms that define them.

### 5.2. Addiction

Addiction is a charged and complex term. How it is defined and the connotation of the term breed complexity. Addiction encompasses a large number of disorders of over-consumption—all of which are chronic and relapsing and span neurological and psychological fields. Addiction costs the United States more than $740 billion per year in healthcare costs, crime, and lost productivity [146]. Drug overdose deaths surpassed 100,000 in the United States this last year [147]. Combined with the 88,000 annual deaths related to excessive alcohol use and the estimated 480,000 tobacco-related deaths annually, addiction is a leading cause of both mortality and systemic strain in the United States [148].

For this review, we will consider addiction in terms defined by the Diagnostic and Statistical Manual, fifth edition (DSM-V). The DSM-V uses the term “substance use disorders” rather than addiction, but in the current context, the two are synonymous. The category encompasses ten classes of drugs and is defined by eleven criteria. Briefly, these criteria include: taking the substance more than intended; wanting to stop but failing to; cravings, tolerance, and withdrawal; spending large amounts of time getting, using, or recovering from the substance; substance use disrupting work, important activities, or relationships; and continuing to use when it is dangerous or causing physical or psychological problems [149].

#### 5.2.1. Neural Mechanisms of Addiction

Numerous social and biological factors affect an individual’s propensity to have a substance use disorder. These include family history, early exposure, social pressures, socioeconomic status, and personal medical history. Recent research has identified neurological factors that play key roles in the development and maintenance of addiction. When considered in its entirety, the variety of substances and their varied mechanisms complicate addiction. However, despite the variety of primary targets for substances of abuse, the neural circuitry involved is largely conserved. The hippocampus, habenula, and amygdala are involved in the strong memories and emotions surrounding drug use and addiction [150]. These brain regions are responsible for the positive feelings of taking a substance as well as the negative emotional states and stress related to withdrawal and negative affect. Of particular importance for this review is the mesolimbic pathway, the brain’s reward circuit. This is a dopamine-dependent circuit that responds to rewards and guides behavioral responses to them. Substances of abuse cause a dopamine release that drives drug wanting [150,151].

The mesolimbic pathway connects the ventral tegmental area (VTA) to the nucleus accumbens which then flows to the ventral pallidum [150]. Approximately one-third of neurons in the VTA are GABAergic. The GABAergic neurons of the VTA inhibit other GABA neurons as well as dopamine neurons [152]. These neurons provide the inhibitory tone from the mesolimbic pathway and send inhibitory projections to other regions of the brain. Finally, top-down control of the reward and motivation circuitry comes from the anterior cingulate gyrus and the prefrontal cortex [150]. This circuit computes excitatory and inhibitory activity and sends a signal to the orbitofrontal cortex, the final arbitrator in deciding whether to use the substance in question.

The activity in these circuits is unbalanced in addiction. Both clinical and preclinical studies demonstrate that blunted dopamine signals are associated with decreased reward sensitivity and increased drug use. Drugs of abuse modify activity in these circuits, driving plasticity in the brain; motivation to use drugs is stronger, drug-related memories are more salient, and top-down control of impulses is weaker, summing to compulsive substance use despite detrimental consequences.

#### 5.2.2. Ionic Plasticity in Addiction

Research has now identified a common neurobiological change in the known neurocircuitry of addiction, ionic plasticity. In models of substance use and abuse, alterations in GABA-dependent inhibition have been found in the mesolimbic pathway, particularly within the VTA. KCC2 is not expressed on dopamine neurons in the VTA, suggesting that the downregulation of KCC2 affects GABA neurons almost exclusively [153]. Altered KCC2 dysregulates Cl^−^ concentrations and causes GABA-A receptors to yield excitatory ion currents. Early evidence that ionic plasticity contributes to addiction was provided by Laviolette et al., 2004. They demonstrated that opiate dependence and withdrawal altered a subset of GABAergic neurons in the VTA, causing them to switch from inhibitory to excitatory [152]. This excitatory GABA activity drives increases in GABA release from VTA GABAergic neurons, which reduces dopamine release [154]. Compensating for this reduction would require additional drug intake, fueling the spiral of addiction. Addiction to nicotine, alcohol, opiates, and stimulants have all been linked to altered Cl^−^ homeostasis.

Taylor and colleagues assessed the role of altered GABAergic function in the reward circuitry following chronic opiate exposure and withdrawal. Using a place preference paradigm to assess reward experience, they found that opiate dependence eliminated cocaine place preference [155]. This suggests that opiate dependence blunts the dopamine response triggered by cocaine administration. When they assessed the state of Cl^−^ flux in the VTA, they found altered Cl^−^ transport in VTA GABAergic neurons. A downregulation of KCC2 in the VTA was also confirmed [155]. Taylor and colleagues went on to demonstrate that microglia are activated during opiate withdrawal. These active microglia release BDNF, a known regulator of KCC2, into the VTA, likely causing the KCC2 downregulation. Moreover, KCC2 levels were restored with a TrkB function-blocking antibody further implicating the BDNF cascade in this downregulation [155]. To confirm that ionic plasticity is sufficient to alter the response to cocaine, KCC2 activity was inhibited in opiate naïve rats by administration of furosemide. Here too, cocaine place-preference was eliminated just as it was in opiate-dependent animals [155]. Their results suggest that BDNF plays a pivotal role in the downregulation of KCC2 functionality and that a reduction in KCC2 is sufficient to alter reward processing and drive addictive behavioral phenotypes.

Given that behavioral indicators of addiction are altered by ionic plasticity, researchers explored whether this process plays a role in factors that impact the risk of addiction. A known predisposing factor to substance use disorders is stress exposure. To interrogate the neurobiological mechanisms of this phenomenon, Ostroumov and colleagues examined the effects of stress exposure on alcohol self-administration and the reward circuit. Acute stress exposure yielded a functional downregulation of KCC2 in the VTA [38]. This KCC2 downregulation drove increased GABA neuron excitation in response to alcohol, increasing GABA inhibitory output in the VTA and blunting the dopamine release following alcohol exposure. Importantly, pharmacological inhibition of stress hormones using RU-486, or enhancing KCC2 activity with CLP290, prevented increased alcohol self-administration and restored alcohol-induced dopamine responses [38]. The results suggest that stress increases alcohol consumption because the consequent ionic plasticity blunts dopamine release. Compensation requires increased alcohol consumption.

Given that one risk factor for substance use disorders is driven by ionic plasticity in the reward pathway, it follows that others might be as well. Because tobacco smoking is an established risk factor for subsequent substance abuse, Vihavainen and colleagues examined the effects of nicotine on the mesolimbic circuitry. They demonstrated that chronic nicotine affects neurological responses to morphine, showing that morphine caused a greater release of dopamine 24 h after chronic nicotine administration was stopped compared to nicotine naïve animals [156]. No changes were identified in dopamine synthesis or depletion; however, altered GABA functionality was identified. They suggested that nicotine altered GABAergic control of dopaminergic neurons.

Further evidence that nicotine exposure affects GABAergic processes was provided by Doyon and colleagues [157]. They found that nicotine exposure causes an increase in alcohol self-administration and blunts the dopamine response to alcohol. This blunted reward response to alcohol was attributed to an increase in inhibitory signals to VTA dopaminergic neurons [157]. They then sought to identify a higher order mechanism. Because nicotine increases corticosterone levels, they chose to systemically block glucocorticoid receptors using RU-486. Pretreatment with RU-486 corrected dopamine responses to alcohol, and prevented the increase in alcohol self-administration, making nicotine-exposed rats indistinguishable from control rats [157]. Here again, increases in the inhibitory tone of the VTA and the consequent reduction in dopamine responses to addictive substances were implicated.

Based on the clinical observation that benzodiazepine abuse occurs more often in smokers, Ostroumov and colleagues sought to address the neurobiology behind this predisposition. Ostroumov et al., 2020 demonstrated that the state of KCC2 and thus, GABA inhibition, are rapidly altered by exposure to nicotine [158]. They found that a single nicotine exposure and volitional consumption downregulate KCC2, diminishing Cl^−^ extrusion capacity, which dysregulates the GABA-A receptor activity on GABA neurons in the VTA [158]. As a result, diazepam, a commonly used benzodiazepine, drives VTA GABAergic neurons toward paradoxical excitation, increasing GABAergic inhibition of dopaminergic neurons and reducing dopamine release to diazepam administration. Furthermore, these effects were lasting, seen fifteen hours after the nicotine exposure. Behavioral data confirmed that the neurobiological changes had a functional effect. A single exposure to nicotine drove an increase in diazepam self-administration. Furthermore, the effects of nicotine exposure could be prevented by enhancing KCC2 activity. By administering CLP-290, Ostroumov and colleagues were able to correct diazepam-induced alterations in GABA activity in the VTA and reduce diazepam self-administration [158].

Taken together, these data demonstrate that nicotine exposure drives ionic plasticity in the reward circuit. This shift then blunts dopamine release secondary to administration of other substances of abuse. Thus, nicotine sets the brain up for addiction by way of ionic plasticity.

Another risk factor for substance abuse is drug exposure in adolescence. Thomas and colleagues shed a unique light on this issue as it relates to ionic plasticity. They administered nicotine to both adolescent and adult rats and examined alcohol self-administration later in adulthood as well as GABA transmission, Cl^−^ homeostasis, and KCC2 expression [159]. Interestingly, they found that nicotine exposure in adolescents, but *not* in adulthood, induces a lasting increase in alcohol self-administration. This increase was accompanied by long-term alterations in the currents through GABA-A channels and downregulation of KCC2 on VTA GABAergic neurons [159]. These changes result in altered reward signaling in the VTA by increasing the inhibition of dopamine neurons, attenuating the response to alcohol. Thomas et al., 2018 took these findings a step further, aiming to correct the changes in Cl^−^ homeostasis seen in adolescent nicotine exposure. By doing so, they found that alcohol self-administration returned to control levels [159]. These data provide a neurological mechanism for nicotine as a gateway drug and identify adolescence as a particularly vulnerable period.

### 5.3. Summary

The findings reviewed above provide evidence that ionic plasticity contributes to disease states in the brain as well as the spinal cord. Impaired GABAergic inhibition leads to overexcitation in the brain that can manifest in different forms depending on the brain region affected. Global effects yield seizures and epilepsy. Altered timing of GABAergic development contributes to neurodevelopmental diseases and ionic plasticity within reward pathways can foster addiction. These brain-mediated pathologies share the clinical feature of being extremely difficult to treat. The identification of ionic plasticity as a shared mechanism opens new avenues for therapeutics.

## 6. Other Disease States

The scope of ionic plasticity reaches even further. While the role of ionic plasticity in a number of neurological disease states is clear, alterations in GABA function have been shown to contribute to numerous disease states that are primarily characterized by their effects on other organ systems. These include hypertension, asthma, and irritable bowel syndrome.

### 6.1. Hypertension

Hypertension, a well-known “silent killer”, affects nearly 50% of adults in the United States (45.4% of adults) and is a major risk factor for other diseases as well [160]. The etiology of essential hypertension is poorly understood. Work by Ye and colleagues suggests that ionic plasticity is involved in the development of hypertension [161]. Using spontaneously hypertensive rats, they demonstrated that altered GABA activity in the hypothalamic paraventricular nucleus (PVN) caused increased sympathetic drive which increased blood pressure [161]. The removal of tonic GABAergic inhibition in the PVN was the result of increased NKCC1 and highly active N-glycosylated NKCC1. KCC2 levels, on the other hand, did not differ between control and hypertensive rats [161]. The findings suggest that hypertension can be driven by ionic plasticity, which alters the excitatory-inhibitory balance of the sympathetic nervous system.

Kim and colleagues extended these observations, demonstrating that risk factors for hypertension can drive ionic plasticity [162]. They assessed the inhibitory-to-excitatory switch of GABA in the development of sodium-dependent hypertension. Like Ye and colleagues, Kim et al. identified altered GABA functionality in the PVN. Specifically, they found that angiotensin-vasopressin-producing neurons had altered Cl^−^ homeostasis that resulted from the upregulation of NKCC1 and the downregulation of KCC2 [162]. GABA, therefore, became excitatory in the angiotensin-vasopressin-producing neurons, driving more vasopressin release causing vasoconstriction and, consequently, hypertension. Taken together, these studies demonstrate that ionic plasticity can promote hypertension and suggests that therapeutic intervention targeted at restoring Cl^−^ balance might be an ideal candidate for antihypertensives.

### 6.2. Asthma

He and colleagues examined the role of neuronal Cl^−^ homeostasis in asthma [163]. It is known that vagal hypertonia is closely related to the severity of asthma as it controls airway smooth muscle. The mechanism behind this hypertonia, however, was unclear. They found increased microglial activation in the brainstem, particularly in the nucleus accumbens [163]. He et al. then identified an upregulation of NKCC1 and downregulation of KCC2 in the nucleus accumbens, which would augment the excitatory vagal response. Injected GABA caused an excitatory response leading to airway hyperresponsiveness that was attenuated by injection of bumetanide, the NKCC1 inhibitor [163]. Wang and colleagues assessed the efficacy of furosemide treatment in allergic asthma and in the process identified increased NKCC1 immunoreactivity in the lungs of an asthmatic mice model [164]. Together, these findings give a mechanistic basis to therapeutic studies demonstrating that furosemide and bumetanide relieve asthma in both animal models [165] and human patients [166].

### 6.3. Irritable Bowel Syndrome

Irritable bowel syndrome affects up to 30% of the worldwide adult population [167]. One of the key components of irritable bowel syndrome is visceral hypersensitivity characterized by reduced pain threshold and exaggerated response to colorectal distention. To elucidate the mechanisms behind this Tang et al., assessed the role of KCC2 in chronic stress-induced visceral hypersensitivity [168]. They found that KCC2 was significantly reduced in the spinal cord segments that innervate the colon (L6-S1). NKCC1 levels were unaffected in rats with visceral hypersensitivity [168]. Functional KCC2-mediated Cl^−^ extrusion capacity was also impaired. Furthermore, blockade of KCC2 was sufficient to induce a rapid and reversible visceral hypersensitivity [168].

### 6.4. Summary

The reach of ionic plasticity spans numerous organ systems. While not traditionally characterized as neurological diseases, the diseases discussed in this section of the review demonstrate how neurological malfunction can adversely affect end organ function. Evidence from hypertension, asthma, and IBS demonstrate that ionic plasticity leads to hyperactivity in whatever nerve or segment of the nervous system is affected. This overexcitation can then yield symptoms in the organs innervated by the affected segment. Further research will likely identify other instances of ionic plasticity driving disease states.

## 7. Conclusions

The nervous system requires a balance of excitatory and inhibitory activity to yield meaningful signals that carry information to and from the effector organs. As the major inhibitory neurotransmitter of the mature CNS, GABA’s essential role in this balance has been long recognized. It is now clear that the traditional paradigm of GABA’s functionality as solely inhibitory fails to encompass its full range of function. The effects of GABA are far more flexible than originally believed.

It has been known for decades that the regulation of intracellular Cl^−^ concentrations can alter how engaging the ionotropic GABA-A receptor affects neural activity. In early development and mature primary afferent neurons, there is a high intracellular concentration of Cl^−^. In both cases, this occurs because cells express the co-transporter that moves Cl^−^ into the cell (NKCC1), but lack the co-transporter required for extrusion (KCC2), leading to an accumulation of Cl^−^ within the cell [2,89]. Under these conditions, engaging GABA-A allows Cl^−^ to exit the cell, which has a depolarizing effect. Ben-Ari and colleagues saw the maturation of KCC2, and the shift in how GABA affects neural activity, as an essential developmental step—that promotes circuit formation in early development [18,169]. This shift would yield a less malleable adult CNS, with GABAergic inhibition functioning to preserve neural circuits over time.

The work reviewed here stems from the recognition that processes beyond development can dynamically regulate intracellular Cl^−^ concentrations, thereby altering how GABA affects neural activity in the mature CNS. It is assumed that this process, ionic plasticity, normally subserves an adaptive function, removing a GABA-dependent brake on neural activity to promote plasticity in a particular neural circuit. From this perspective, ionic plasticity is required for learning [170]. Indeed, bumetanide has been shown to disrupt the development of hippocampal LTP in vitro and inhibitory avoidance learning in vivo [171]. Further increases in excitability could saturate NMDA-Receptor mediated plasticity and undermine learning about specific relations (Figure 3D). Given these observations, we have suggested that GABA-dependent inhibition limits the scope of neural activity whereas the removal of this brake broadens the response profile [170]. Likewise, others have proposed that compromised inhibition reduces the fidelity of information transfer, leading to a loss of pathway-specific LTP [26,172]. However, in response to neural injury, inflammation, or disease, a pathological rise in intracellular Cl^−^ concentrations can develop that enables neural over-excitation. As we have seen, these effects are evident after SCI, where ionic plasticity enables aberrant motor activity and the sensitization of nociceptive circuits. In part, these effects may emerge because there is a lessening of the GABA-dependent brake on neural activity. However, in some instances [e.g., after complete spinal transection and in diabetic neuropathy] [91,124], blocking the GABA receptor with bicuculline prevents nociceptive sensitization, which suggests GABA provides part of the driving (excitatory) force required to initiate NMDAR-mediated plasticity. The latter would modify excitatory glutamatergic synapses, laying down a kind of pain memory that could maintain chronic pain [74,75]. Here, re-establishing low intracellular Cl^−^ concentrations, using treatments that target ionic plasticity, would block the development of nociceptive sensitization but have little effect on its maintenance (after the pain memory has been formed).

A key issue is whether the concept of ionic plasticity has broader implications, that extend beyond an explanation of pathology after SCI. One indication that it does stems from work examining the development of neuropathic pain, which has been related to inflammatory processes (microglia activation) within the spinal cord, leading to BDNF release and a downregulation in KCC2 in the absence of SCI [108,173,174]. Other work has shown that a rise in intracellular Cl^−^ concentrations contributes to seizure activity and addiction by modifying activity in various brain regions [132,152,155]. Beyond this, ionic plasticity has been implicated in a host of pathologies, including hypertension, asthma, and irritable bowel syndrome [161,162,163,168].

It has been known for many years that treatments that target GABA receptors can bring therapeutic benefits to a range of pathologies. The shared difficulty is that drugs (e.g., benzodiazepines) that broadly augment GABAergic inhibition generally have a sedative effect that interferes with function. Treatments that target ionic plasticity should have a more selective effect, to re-establish GABAergic inhibition in neural regions where intracellular Cl^−^ concentrations are high while having little effect on other parts of the CNS. The one caveat concerns the presumed role of ionic plasticity in adaptive learning [170]. To the extent that a lessening of GABAergic inhibition is required for learning, treatments that generally lower intracellular Cl^−^ concentrations may impair this process. The ultimate goal, therefore, is to find the “happy medium” along the continuum of ionic plasticity where maladaptive plasticity can be avoided, and adaptive plasticity can be harnessed [19].

The recognition that GABA does not always have an inhibitory effect informs the consideration of alternative therapeutics. For example, treatment with a GABA-A agonist, or locally increasing GABA concentrations by implanting cells within the spinal cord that express this transmitter, will not quiet nociceptive circuits when intracellular Cl^−^ concentrations are high (and in some cases could have an adverse excitatory effect). One alternative is to target metabotropic GABA-B receptors. This has proven effective for the treatment of spasticity but has limited applicability for other conditions because these receptors are generally not expressed outside of the spinal cord [1]. Targeting ionic plasticity may bring therapeutic benefits along the neural axis and beyond.

A caution in application stems from the observation that some processes that impact ionic plasticity can have divergent effects. For example, BDNF has been shown to up-regulate KCC2 and attenuate both nociceptive sensitization and spasticity after SCI [3,97]. However, in the absence of neural injury, the neurotrophin appears to downregulate KCC2 and promote pathological over-excitation [108]. Interestingly, this adverse effect does not appear to arise in response to exercise, which generally fosters GABAergic inhibition through a BDNF-dependent process over a range of injury levels [42], implying a form of autoregulation that promotes adaptive plasticity [41]. Another caution stems from recent work showing that ionic plasticity may sometimes play a greater role in males than females [117]. Further work is needed to delineate the circumstances under which targeting ionic plasticity will bring therapeutic benefit.

While a range of experimental tools has helped to elucidate the role of ionic plasticity in pathology and the underlying cellular mechanisms, our focus has been on pharmacotherapies. The reason is that pharmacology offers the most immediate hope for clinical translation. While a strong empirical foundation suggests targeting ionic plasticity has therapeutic potential, clinical trials have been limited. The one exception is epilepsy where it has been shown that bumetanide reduces seizure activity [139,140,141]. In addition, positive results have been reported for pain after SCI and asthma [100,166]. Further work is also needed to evaluate clinical safety, given some potential treatments (e.g., bumetanide) can have adverse secondary effects [175,176]. This is particularly true for drugs that target NKCC1, which is expressed in many tissue types throughout the body [176]. In the long term, agents that target KCC2 should bring therapeutic benefits with fewer secondary side effects. Importantly, new work has identified additional compounds that act as KCC2 expression enhancers [177]. At present, CLP290, which has improved pharmacokinetics relative to CLP257 [178], can be administered orally [45], and does not produce obvious side effects, appears to be the most promising therapeutic option.

The scope of ionic plasticity stretches farther than the pathologies reviewed here. Not only has ionic plasticity been identified in addiction, pain, diabetes, spasticity, seizures, and neurodevelopmental disorders, but in executive function, reward processing, and motivation; anxiety, depression, autism, schizophrenia, Parkinson’s disease, and human cancer cell lines [134,145]. Undoubtedly, ionic plasticity will be implicated in other disease states in the future.

## Data Availability

Not applicable.
